# Elucidating the Contribution of Skeletal Muscle Ion Channels to Amyotrophic Lateral Sclerosis in search of new therapeutic options

**DOI:** 10.1038/s41598-019-39676-3

**Published:** 2019-02-28

**Authors:** Giulia Maria Camerino, Adriano Fonzino, Elena Conte, Michela De Bellis, Antonietta Mele, Antonella Liantonio, Domenico Tricarico, Nancy Tarantino, Gabriella Dobrowolny, Antonio Musarò, Jean-Francois Desaphy, Annamaria De Luca, Sabata Pierno

**Affiliations:** 1 Department of Pharmacy-Drug Sciences, University of Bari Aldo Moro, 70125 Bari, Italy; 2 DAHFMO-Unit of Histology and Medical Embryology, Sapienza University of Rome, 00161 Laboratory affiliated to Istituto Pasteur Italia – Fondazione Cenci Bolognetti, Rome, Italy; 3 Center for Life Nano Science at Sapienza, Istituto Italiano di Tecnologia, 00161 Rome, Italy; 4 Department of Biomedical Sciences and Human Oncology, University of Bari Aldo Moro, Polyclinic, 70124 Bari, Italy

## Abstract

The discovery of pathogenetic mechanisms is essential to identify new therapeutic approaches in Amyotrophic Lateral Sclerosis (ALS). Here we investigated the role of the most important ion channels in skeletal muscle of an ALS animal model (MLC/SOD1^G93A^) carrying a mutated SOD1 exclusively in this tissue, avoiding motor-neuron involvement. Ion channels are fundamental proteins for muscle function, and also to sustain neuromuscular junction and nerve integrity. By a multivariate statistical analysis, using machine learning algorithms, we identified the discriminant genes in MLC/SOD1^G93A^ mice. Surprisingly, the expression of ClC-1 chloride channel, present only in skeletal muscle, was reduced. Also, the expression of Protein Kinase-C, known to control ClC-1 activity, was increased, causing its inhibition. The functional characterization confirmed the reduction of ClC-1 activity, leading to hyperexcitability and impaired relaxation. The increased expression of ion channel coupled AMPA-receptor may contribute to sustained depolarization and functional impairment. Also, the decreased expression of irisin, a muscle-secreted peptide protecting brain function, may disturb muscle-nerve connection. Interestingly, the *in-vitro* application of chelerythrine or acetazolamide, restored ClC-1 activity and sarcolemma hyperexcitability in these mice. These findings show that ion channel function impairment in skeletal muscle may lead to motor-neuron increased vulnerability, and opens the possibility to investigate on new compounds as promising therapy.

## Introduction

Amyotrophic Lateral Sclerosis (ALS) is a progressive degenerative disease affecting motor neurons. Because of disrupted nerve-muscle communication, afflicted individuals progressively lose control of voluntary muscle function and experience muscle weakness until paralysis. Many of the familial cases of ALS are due to mutations within the gene encoding the superoxide dismutase 1 (SOD1) protein, involved in the detoxification of reactive oxygen species^1^. Despite many advances in the understanding of the genetic causes of ALS, there is no effective treatment available for this devastating disease. It is thus imperative to gain insights in the pathophysiology of ALS. It is recognized that ALS involves tissues other than nerves. Skeletal muscle is one of the earliest impaired tissues in ALS, with fasciculation, force decrease, and atrophy^2^. Indeed, skeletal-muscle-restricted expression of mutant SOD1 gene in MLC/SOD1^G93A^ mice causes progressive muscle atrophy, with a significant reduction in muscle strength, alterations in the contractile apparatus, and mitochondrial dysfunction^3–5^. The analysis of molecular pathways revealed that accumulation of oxidative stress triggers intracellular degradation mechanisms. Moreover, alterations of the neuromuscular junction (NMJ) seems to play a significant role in disease progression^6^. Indeed, the very earliest manifestation of disease in both the SOD1^G93A^ mouse model (carrying G93A mutation in SOD1 protein) and ALS patients occurs at the NMJ, where significant levels of denervation can be observed before the onset of motor neuron degeneration^7^. The analysis of molecular mechanisms involved in NMJ dismantlement revealed a link between Protein Kinase C-theta (PKC-theta) activation and NMJ disintegration^4^. Conversely, trophic factors secreted by myofibers, such as Insulin Like Growth Factor-1 (IGF-1) or Glial-Cell-Line-Derived Neurotrophic Factor (GDNF), can promote motor neuron survival in ALS model through stabilization of NMJ^8^. These observations support the view that this pathology is not solely a neurological disorder but also include a “dying-back” phenomenon, by which motor unit loss and altered muscle function precede the death of motor neurons^9^. In this context, sarcolemma ion channels, such as Cl^−^, K^+^, Na^+^ and Ca^2+^ channels play a crucial role in maintaining muscle function. They are involved in the control of muscle excitability, contraction, and plasticity. Mutations in these channels cause inherited channelopathies^10^. For instance, the ClC-1 chloride channel is typically expressed in skeletal muscle and controls resting membrane potential and excitability^11,12^. It sustains the membrane Chloride Conductance (gCl) at rest and its activity is regulated by the PKC-theta, able to phosphorylate and close the channel, and to maintain a low gCl during the first phase of action potential^13^. Hereditary loss-of-function mutations in the ClC-1 channel are responsible for Myotonia Congenita, a disease characterized by impairment of muscle excitability and relaxation^14^. Increased muscle excitability and reduction of gCl are also observed during sarcopenia^15^ or hypolipidemic drug adverse effects^16,17^. The ATP-sensitive potassium (KATP) channels associate muscle cell metabolism and electrical activity, they play an important role in the control of contractility, particularly when cellular energetic is compromised, protecting the tissue against calcium overload and fiber damage. Because ion channels activity and expression can be modified either directly or indirectly by oxidative stress^15,18,19^, they represent potential targets of ALS pathomechanism. Many studies have focused on the alterations of neuronal excitability in sporadic and familial cases of ALS, due to abnormalities in axonal Na^+^ and K^+^ conductance^20^. It is widely acknowledged that excitotoxicity is an important contributor to ALS by promoting a neurodegenerative cascade via Ca^2+^-mediated processes^21^. Accordingly, the Na^+^ channel blockers, such as mexiletine, have been tested to promote membrane stabilization and to control ALS symptoms^22^. However, there are no reports on ClC-1 channel involvement in ALS, a channel exclusively expressed in skeletal muscle. In the light of these considerations, we focused our study on ion channel expression and function in skeletal muscles of transgenic SOD1^G93A^ mice and in muscle specific MLC/SOD1^G93A^ mice (carrying the SOD1^G93A^ mutant gene under the transcriptional control of Myosin Light Chain, MLC, a muscle-specific promoter) to elucidate the mechanisms and to find potential therapeutic targets in ALS.

## Results

### Gene expression profile in skeletal muscle of SOD1^G93A^ and MLC/SOD1^G93A^ mice

The quantitative Real-Time PCR analysis have been used to detect skeletal muscle alterations due to SOD1 mutation in both SOD1^G93A^ and MLC/SOD1^G93A^ mice with respect to age-matched and strain-matched wild-type (WT). We evaluated the expression of a series of genes involved in skeletal muscle structure and function, encoding for ion channels, their regulatory proteins and subunits, structural proteins, markers of denervation (Supplementary Fig. S1) and phenotype shift (Supplementary Fig. S2), hormones and neurotrophic factors. The involvement of these genes in ALS etiology was evaluated by an accurate multivariate statistical analysis PCA-LDA (Principal Component Analysis and Linear Discriminant Analysis), using both unsupervised and supervised machine learning algorithms, which allowed to identify new potential therapeutic targets.

Expression level of ion channels (ClC-1, Nav1.4, BK, Sur1, Kir6.2, Sur2a, Sur2b), transporters (TauT), pumps (SERCA1, SERCA2), protein kinases (PKC, AMPK), phosphatases (CN), marker of muscle atrophy (Murf1), growth factor (NGF) myokines (irisin, myostatin), and receptors (NMDAR, AMPAR, RyR, Notch1).

**Figure 1:**
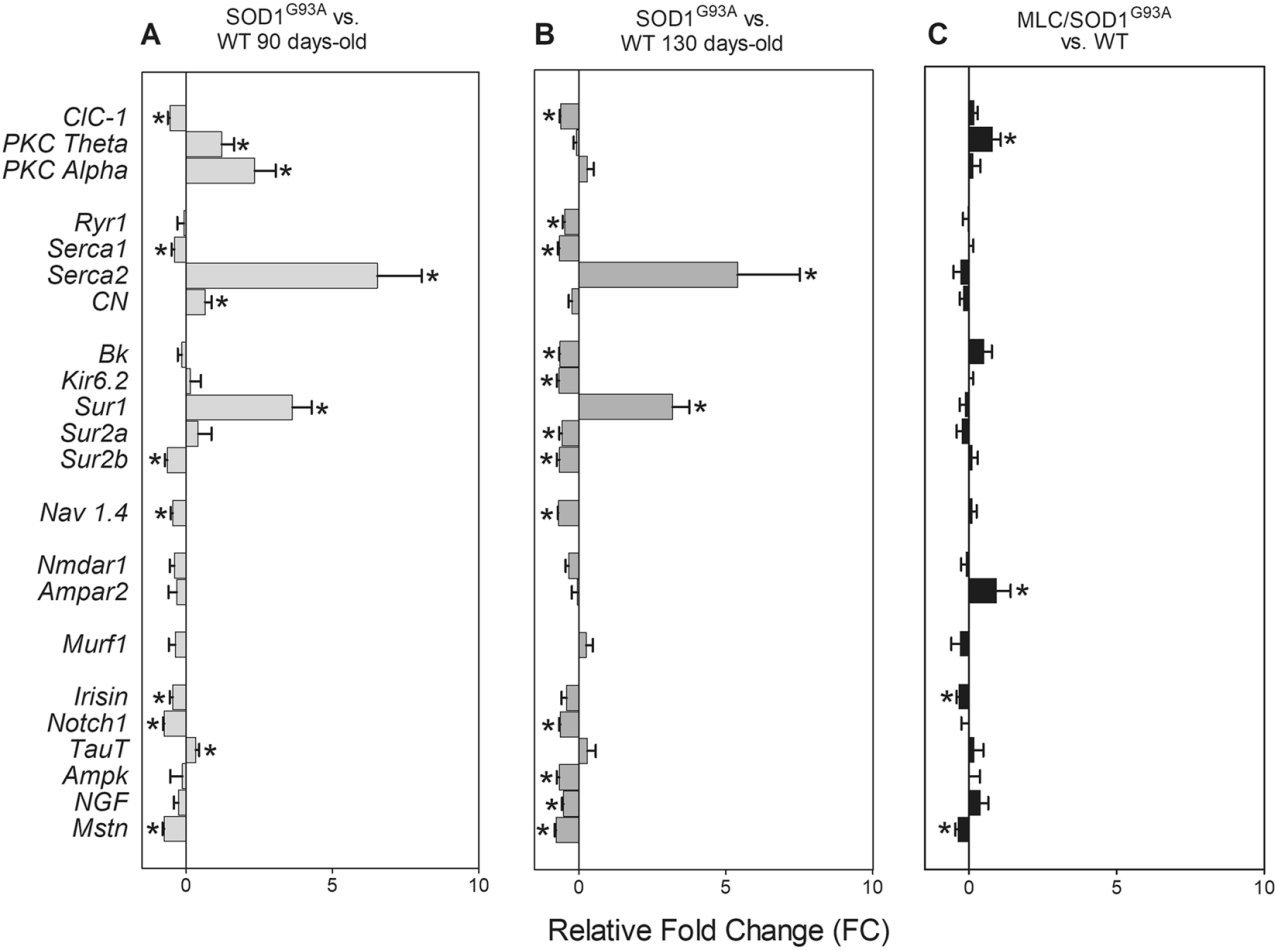
Gene expression changes in SOD1^G93A^ and MLC/SOD1^G93A^ mice. mRNA levels of target genes in Tibialis Anterior (TA) muscles of 90 days-old (**A**), 130 days old (**B**) SOD1^G93A^ and (**C**) MLC/SOD1^G93A^ mice (140 days-old). Data are expressed as Relative Fold Change (FC, calculated as [(Transgenic/ctrl value)-1]) versus the strain- and age-matched controls, of transcript levels performed by Real-Time PCR, for chloride channel ClC1, Protein Kinase C theta (PKC theta), Protein Kinase C alpha (PKC alpha), Ryanodine Receptor 1 (Ryr1), SERCA1, SERCA2, Calcineurin (CN), BK, Kir6.2, Sur1, Sur2, Sur2a, Sur2b, Nav1.4, Nmdar1, Ampar2, Murf1, Irisin, Notch1, TauT, Ampk, Nerve Growth Factor (NGF), Myostatin (Mstn) normalized by β-Actin housekeeping gene, in the 6 experimental groups. Each bar represents the FC ± S.E.M. Five muscles for each experimental group were analyzed and each muscle was analyzed in triplicate.

In Tibialis Anterior (TA) muscles of SOD1^G93A^ mice at 90 days of age, we found a significant down-regulation of mRNA level of ClC1 chloride channel, potassium channel subunit Sur2b, sodium channel subunit Nav1.4, calcium pump SERCA1, the muscle-secreted hormone irisin, myostatin (Mstn), and Notch1, involved in satellite cell proliferation, with respect to that found in age-matched WT mice (Fig. 1). The mRNA level of PKC-theta, PKC-alpha, SERCA2, Calcineurin (CN), Sur1, and the taurine transporter TauT, was significantly up-regulated with respect to that measured in the age-matched WT animals (Fig. 1). In TA of 130 days-old SOD1^G93A^ mice, a higher number of genes was down-regulated, including ClC1, SERCA1, Big Potassium Channel (BK), major subunit of ATP-sensitive inward-rectifier potassium ion channels (Kir6.2), Sulfonylurea receptors 2a and 2b (Sur2a and Sur2b), Nav1.4, AMP-activated protein kinase (AMPK), Ryanodine receptor 1 (Ryr1), Notch1, nerve growth factor (NGF) and Mstn (Fig. 1). Alongside, the mRNA level of SERCA2 and Sulfonylurea receptor 1 (Sur1) was significantly up-regulated (Fig. 1). In TA muscles of MLC/SOD1^G93A^ we found down-regulation of irisin and Mstn and up-regulation of AMPAR2 and of PKC-theta as compared to the strain-matched WT (Fig. 1).

Multivariate Statistical Analysis of gene expression data. Principal Component Analysis (PCA) and Linear Discriminant Analysis (LDA)

**Figure 2:**
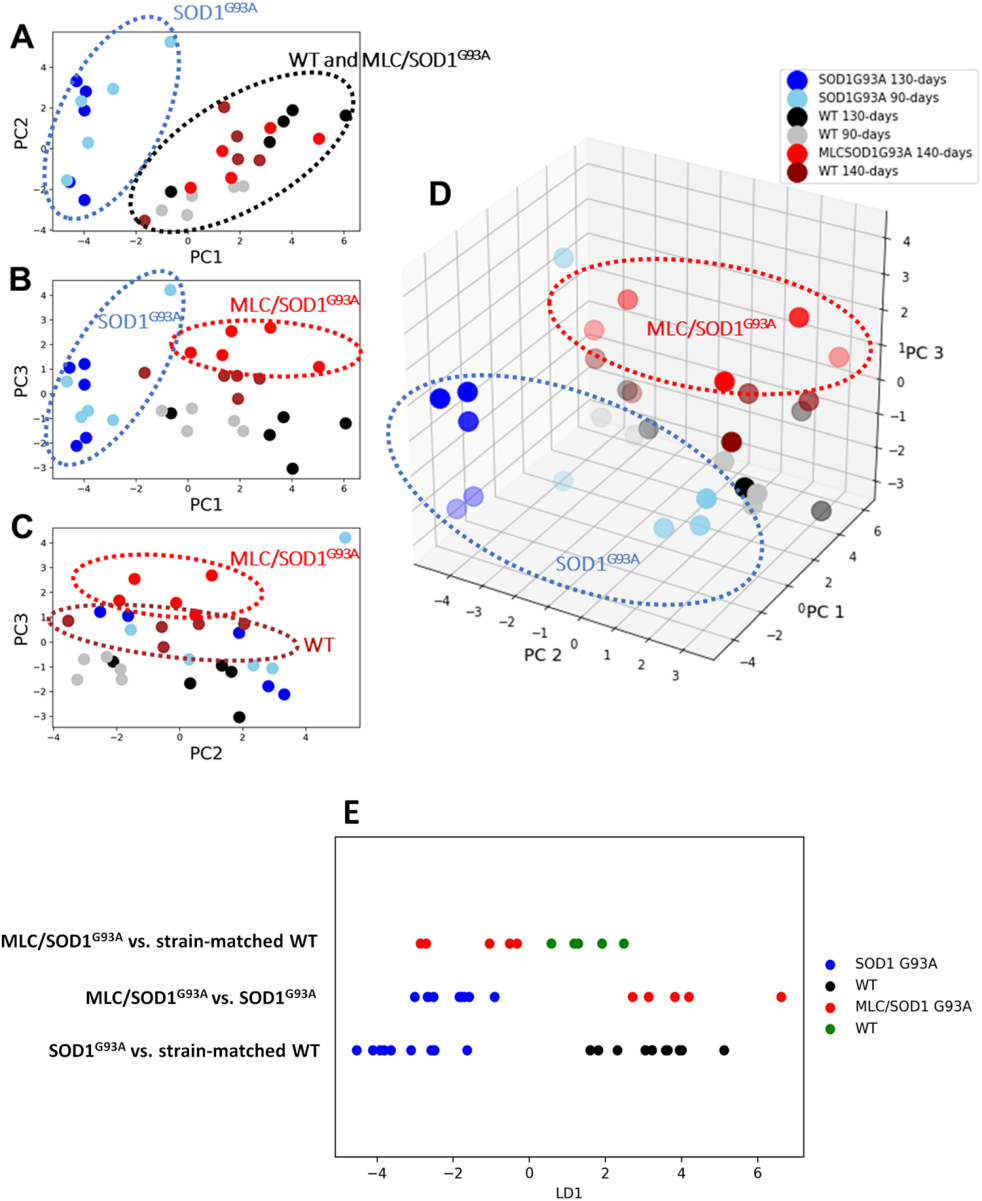
Principal Component Analysis (PCA) and Linear Discriminant Analysis (LDA) of PCA scores. Scatterplot of PC score onto the first three selected PC. In (**A**) are shown PC score on PC1 and PC2, in (**B**) of PC1 and PC3, in (**C**) of PC2 and PC3, as 2-dimensional graphs. In (**D**) a 3-dimensional scatterplot of the same PC scores. Each dot represents the intersection of PC scores of the animal muscles (n = 30) onto PC1, PC2 and PC3. PC score are linear combinations of initial gene expression values of target genes and their corresponding loading weights in the considered PC. (**E**) Linear scatter-plots showing the results of the 3 Linear Discriminant Analysis (LDAs) of the first three PC scores. Red dots are MLC/SOD1^G93A^ muscles Linear Discriminant (LD) scores, green dots are the strain-matched WT LD scores, blue dots are SOD1^G93A^ LD scores and black dots strain-matched WT LD scores. On the top was showed the first LD, that better separates red and green dots representing MLC/SOD1^G93A^ and their WT, respectively. In the middle part, LD that better separates MLC/SOD1^G93A^ and SOD1^G93A^ muscles, shown as red and blue dots, respectively. On the bottom, LD that better separates SOD1^G93A^ and their WT, shown as blue and black dots, respectively.

**Figure 3:**
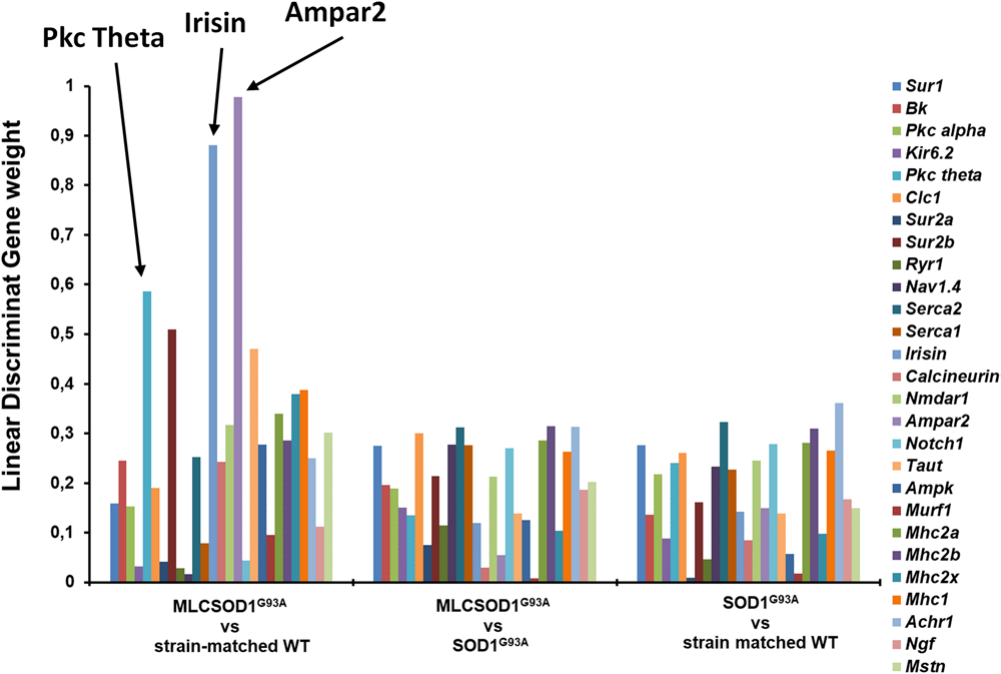
PCA/LDA Discriminant Genes. Barplots of Linear Discriminant loading weights of each gene in the three PCA-LDA analysis performed. Each bar represents the weight of the variable in order to discriminate between: (on the left) MLC/SOD1^G93A^ vs. strain matched WT, (in the middle) MLC/SOD1^G93A^ vs. SOD1^G93A^ and (on the right) SOD1^G93A^ vs. strain-matched WT PCA scores. On the right, a legend showing the colors-code to identify the mRNA LD gene loading weight of the target genes. Arrows indicates the most affected genes.

The gene expression dataset was composed of 27 columns representing the expression level of genes normalized to β-Actin (housekeeping gene) and 30 rows representing the analyzed TA muscles. This dataset has been used to perform a PCA calculating 27 eigenpairs of eingenvector-eigenvalue to project in the newly modelled subspace of characteristics. This analysis shows that the first 3 Principal Components (PCs) accounted for about the 2/3 of the total of the variance (Supplementary Fig. S3, Tables S1 and S2). Muscles of SOD1^G93A^ group are well separated from the others (Fig. 2A–C). The selected genes were able to separate well also the less affected MLC/SOD1^G93A^ group from their WT. These observations were evident after analysis of the scatterplots in Fig. 2D. In order to confirm it mathematically, we used the PCA-LDA approach. Thus, training an LDA algorithm, we obtained the contribution of each PC to separate the three pairs of groups: (1) SOD1^G93A^ and their age-and strain-matched controls; (2) MLC/SOD1^G93A^ and their strain-matched controls; (3) MLC/SOD1^G93A^ and SOD1^G93A^. The three LDA (Fig. 2E) were able to find directions (Linear Discriminant, LD) that well segregate samples. The LD coefficients indicated the importance of each PC to separate groups (Supplementary Table S3). Through linear combination of LD coefficients and the PC weight of each gene transcripts (PCA-LDA pipeline), we computed three lists of discriminant genes (Fig. 3, Supplementary Table 4) showing a high weight in the distinction between groups. Farther from the zero the weight is, the greater its discriminating ability. For the separation of MLC/SOD1^G93A^ and WT, the genes with the higher discriminant power were AMPAR2, PKC-theta, Irisin. For MLC/SOD1^G93A^ and SOD1^G93A^ pairs, the highest discriminant genes were Myosin Heavy Chain-2b (MyHC-2B), Acetylcholine Receptor Subunit alpha-1 (AChRa1), SERCA2, ClC1, Myosin Heavy Chain-2a (MyHC-2A), Nav1.4. For separation between SOD1^G93A^ and WT, the following genes were discriminant: AChRa1, SERCA2, MyHC-2B, MyHC-2A, Notch1, Sur1, Myosin Heavy Chain-1 (MyHC-1), ClC-1.

### Hierarchical agglomerative clustering

**Figure 4:**
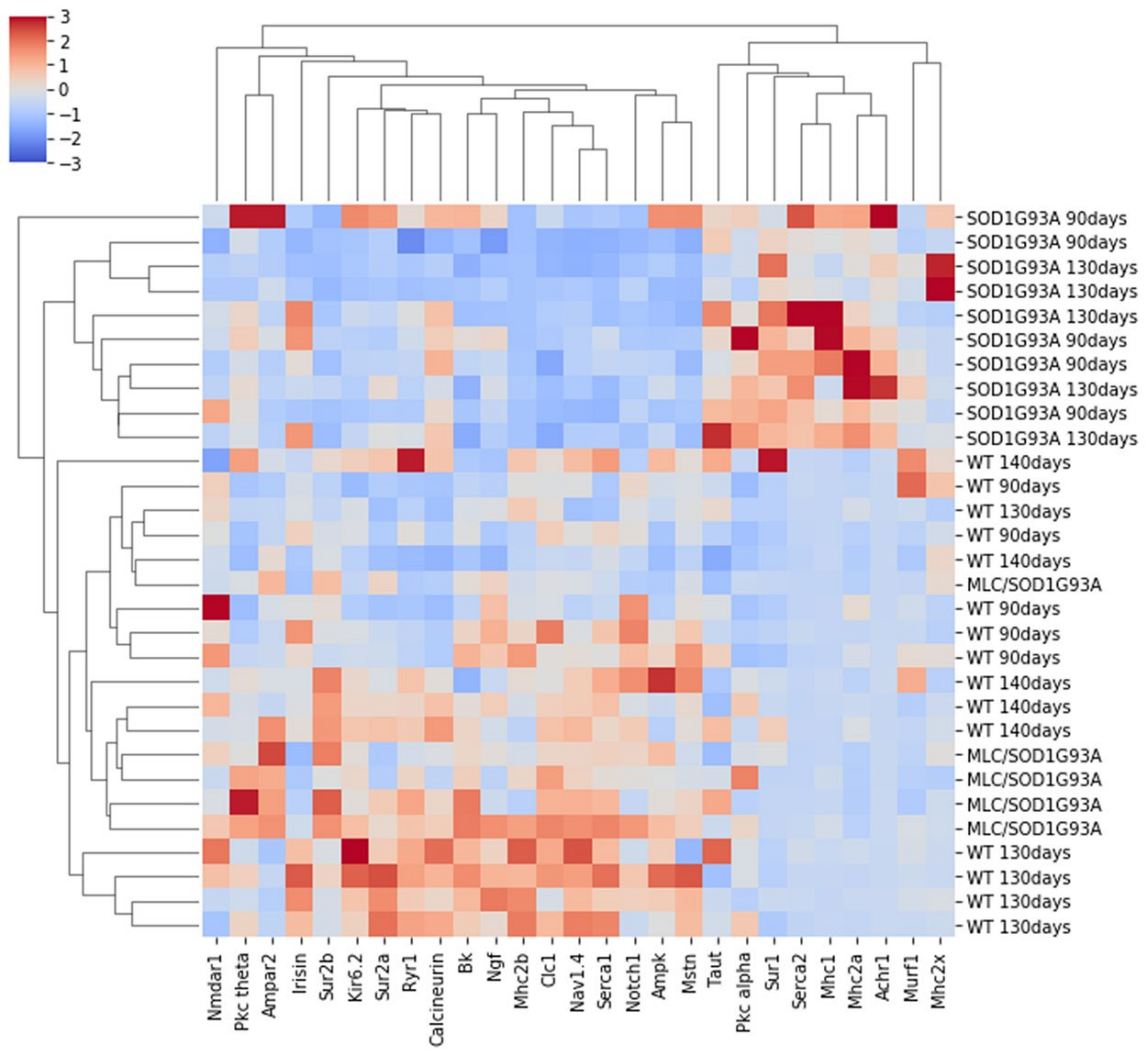
Hierarchical agglomerative clustering of gene expression data. Heatmap of unsupervised machine learning algorithm to perform clusterization of muscles based on gene expression levels. TA muscles and mRNA levels segregated into two principal clusters also in this case, as showed by hierarchical dendrograms of rows and columns. Each square represents the standardized gene expression level normalized with β-actin housekeeping gene in TA muscle of target genes. The columns report target genes, rows report the analysed muscles. Genes and muscles are sorted by hierarchical agglomerative clustering algorithm. Plotted data consists of the entire dataset of animals (SOD1^G93A^, MLC/SOD1^G93A^ and their strain-matched WT as control).

To confirm the pattern modification of transcript levels, we used a hierarchical agglomerative clustering algorithm (Fig. 4). This analysis showed, in an unsupervised manner, that both genes and muscles segregated into two big different clusters. Also in this case, the clusters segregated muscles belonging to ubiquitously SOD1^G93A^ into (MLC/SOD1^G93A^) and WT, highlighting that the major part of the variance is due to variation in gene expression level occurring in SOD1^G93A^ mice.

### ClC-1 protein expression measured by western blot and immunofluorescence assay in SOD1^G93A^ and MLC/SOD1^G93A^ mice

**Figure 5:**
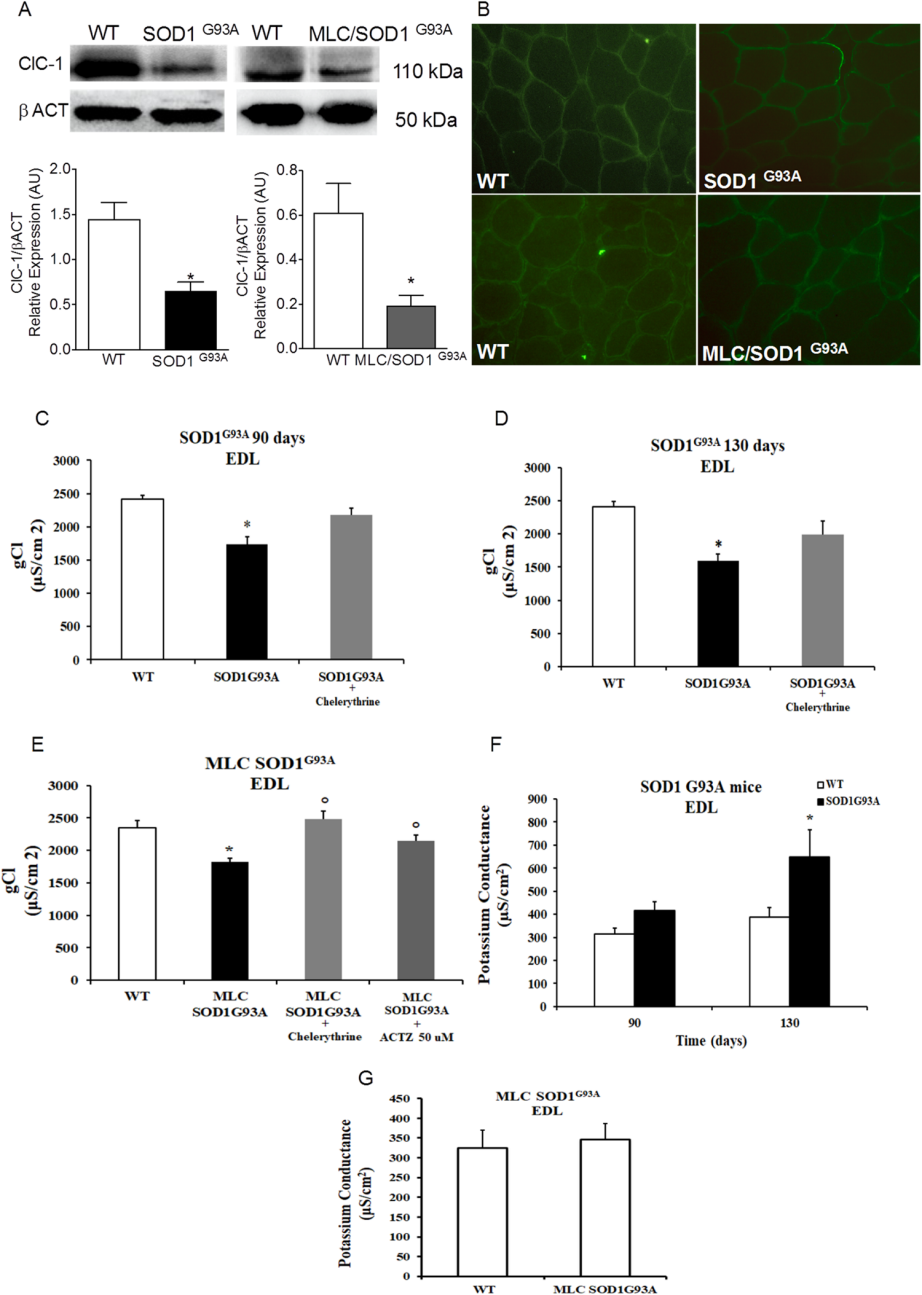
ClC-1 chloride channel expression and activity in MLC/SOD1^G93A^ and SOD1^G93A^ mice. (**A**) Representative Western blot showing the expression level of ClC-1 protein in TA muscle tissue of SOD1^G93A^ and MLC/SOD1^G93A^ mice. The blots were reacted with specific antibodies. β-actin was used to normalize the blot. Gel images are cropped from the blot shown in the Supplementary Figs S7 and S8. Histograms show quantification of relative protein levels calculated by normalization of the absolute intensity of target protein with the absolute intensity of β-actin, as reference standard, and are represented as arbitrary units (AU). Each bar represents the mean ± SEM from five muscles.* Significantly different with respect to WT (at least P < 0.05) by Student’s t-test. (**B**) Immunofluorescence assay in TA muscle of MLC/SOD1^G93A^ and WT mice and in Gastrocnemius muscle of SOD1^G93A^ mice and WT. The presence of ClC-1 protein in muscle section was shown by specific antibodies. The images are 20X magnification. (C-G) The component ionic conductances measured in EDL muscle of SOD1^G93A^ and MLC/SOD1^G93A^ animals. Values are expressed as mean ± S.E.M. from five animals for each experimental condition (10–46 fibers were analyzed in each group of animals). (**C**,**D**) The macroscopic Chloride Conductance (gCl) measured in EDL muscles fibers of SOD1^G93A^ animals at the two selected ages and effects of the *in-vitro* application of chelerythrine (1 µM). Statistical analysis was performed using one-way ANOVA followed by Bonferroni post-hoc t-test (F = 15.8, df = 2, P < 0.0001, for gCl in SOD1^G93A^ at 90-days) (F = 16.6, df = 2, P < 0.0001 for gCl in SOD1^G93A^ at 130-days). *Significantly different vs. age-matched WT (at least P < 0.05). (**E**) Macroscopic gCl measured in EDL muscles fibers of MLC/SOD1^G93A^ animals and effect of *in-vitro* application of Chelerythrine (1 µM) or Acetazolamide (ACTZ, 50 µM). Statistical analysis was performed using one-way ANOVA followed by Bonferroni post-hoc t-test (F = 12.5, df = 3, P < 0.0001, for gCl in MLC/SOD1^G93A^). *Significantly different vs. age-matched WT (at least P < 0.05). °Significantly different vs. MLC/SOD1^G93A^ (at least P < 0.05). (**F**) The macroscopic Potassium Conductance (gK) measured in EDL muscles fibers of SOD1^G93A^ animals at the two selected ages (F = 5.18, df = 3, P < 0.005, for gK in SOD1^G93A^). *Significantly different vs. age-matched WT (at least P < 0.05). (**G**) The macroscopic gK measured in EDL muscle of MLC/SOD1^G93A^ mice (no significant differences were found).

To better explore on the ClC-1 channel modification in skeletal muscle of ALS mice we analyzed the expression of ClC-1 protein by Western Blot (WB) analysis in TA muscles. We found a significant reduction of ClC-1 expression with respect to WT in both models (Fig. 5A), suggesting that a significant less amount of channel protein contributes to the gCl. Since the mRNA level of ClC-1 gene was not significantly changed in the muscle of MLC/SOD1^G93A^ mice, it seems that post-transcriptional events are involved, i.e. an alteration of translation or a rapid degradation of the protein. We also evaluated, by an immunofluorescence assay, the presence of the ClC-1 in cryosections of TA muscle excised from MLC/SOD1^G93A^ mice and of gastrocnemius muscle from SOD1^G93A^ mice (Fig. 5B) both with high expression level of the transgene^2^. The presence of ClC1 protein in muscle sections was shown by using specific antibodies and no modifications were observed in both situations (Fig. 5B).

### Functional characterization of the SOD1^G93A^ and MLC/SOD1^G93A^ mouse models

Resting chloride and potassium conductances in Extensor Digitorum Longus (EDL) muscle of SOD1^G93A^ and of MLC/SOD1^G93A^ mice and effects of *in vitro* application of chelerythrine and acetazolamide

We measured the resting component conductances (gCl and gK: Potassium Conductance) in EDL muscle of both ALS mouse models. In detail, SOD1^G93A^ muscle fibers showed a significant reduction of gCl with respect to their strain- and age-matched controls by −27.8 ± 4.2% and −33.9 ± 3.8%, at 90 and 130 days of age, respectively (Fig. 5C,D). Thus, a reduction of this parameter was already present at clinical onset, and was more severe at the exacerbation of the signs of pathology. A slight increase of gK (by +32.8 ± 11.9%) was found in 90 days-old SOD1^G93A^ animals with respect to their controls, while the difference was significant (by +67.5 ± 27.9%) in 130 days-old SOD1^G93A^ mice (Fig. 5F). In MLC/SOD1^G93A^ mice, we found a significant reduction of gCl by −22.3 ± 3.2% with respect to the WT (Fig. 5E). However, no significant differences of gK were observed (Fig. 5G). No modification was found in gCl and gK measured in slow-twitch SOLEUS (SOL) muscles of SOD1^G93A^ and WT at 130 days of age (Supplementary Fig. S4). To evaluate the involvement of the PKC in the reduction of gCl we tested the effect of chelerythrine, a selective inhibitor of PKC^23^. We found a significant restoration of gCl in EDL fibers of SOD1^G93A^ mice at both ages and of MLC/SOD1^G93A^ mice after *in vitro* application of chelerythrine (Fig. 5C–E). In addition, we tested the effects of acetazolamide (ACTZ), an inhibitor of carbonic anhydrase, which has been previously shown to increase heterologously-expressed ClC-1 channel activity^11,14^. Importantly, the *in vitro* application of ACTZ led to an increase of gCl in EDL muscle fibers of MLC/SOD1^G93A^ mice toward the WT value (Fig. 5E) suggesting potential beneficial effects in ALS models characterized by gCl reduction and hyperexcitability.

### Excitability parameters in EDL muscle of SOD1^G93A^ and MLC/SOD1^G93A^ mice

**Figure 6:**
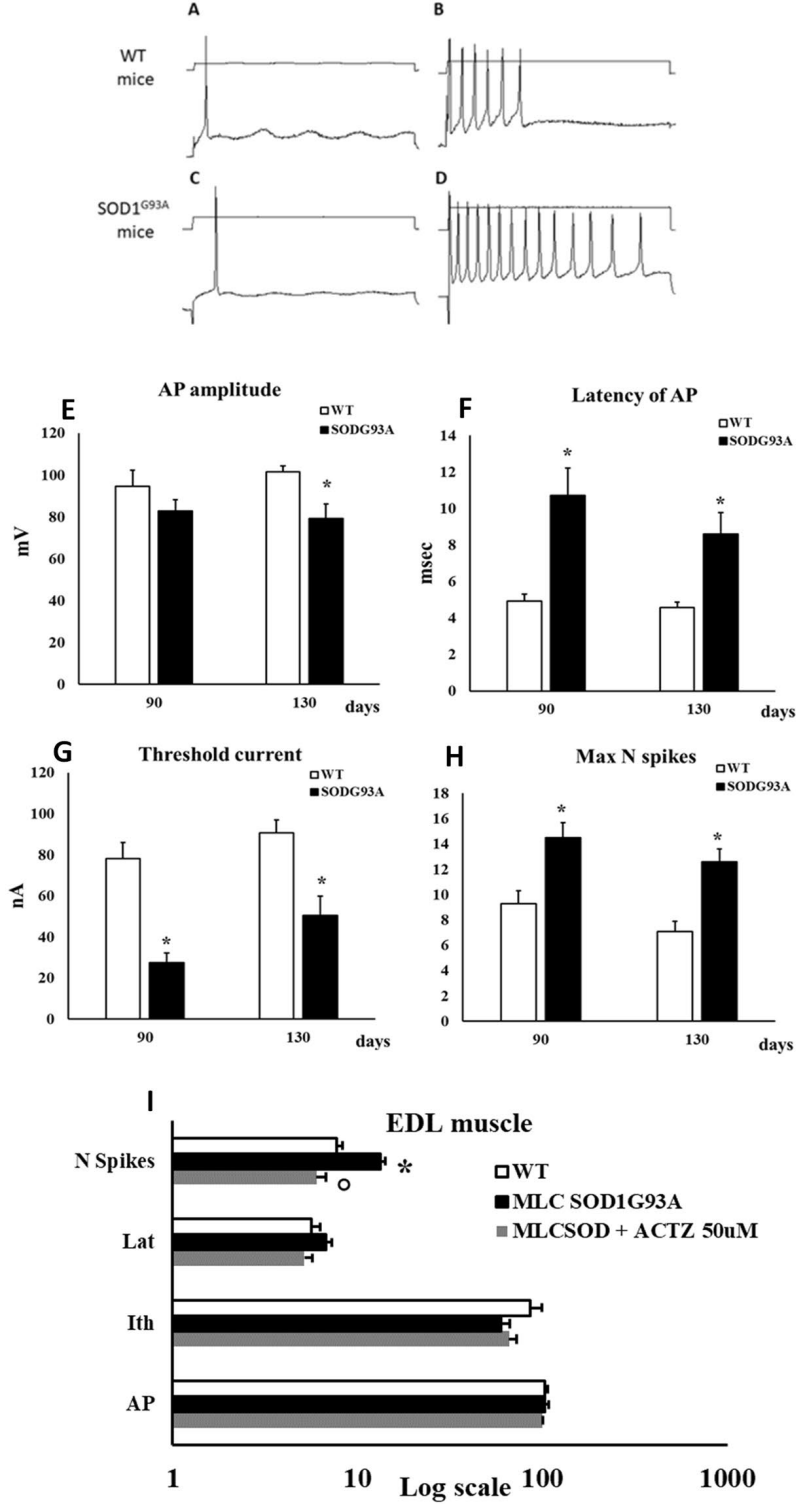
Sarcolemma excitability parameters measured in EDL muscle of SOD1^G93A^ and MLC/SOD1^G93A^ animals. (**A–D**) Representative traces of the Action Potential (AP) recorded in EDL muscle fibers by standard two microelectrodes technique at 0.05 mm distance between electrodes, in response to depolarizing square-wave current pulse. (**A**,**B**) images showed traces recorded in EDL fibers of WT animals. (**C**,**D**) showed the traces recorded in SOD1^G93A^ mice. On the left, it has been used a minimal squared-wave current pulse to elicit the single AP. On the right it has been used the minimum pulse to elicit the maximum number of spikes. (**E–H**) Excitability parameters of EDL muscle fibers of SOD1^G93A^ animals at 90 and 130 days of age. Values are expressed as mean ± S.E.M from 10–16 fibers. In (A) the AP amplitude, in (**B**) the latency time of AP, in (**C**) the threshold current needed to elicit a single AP (Ith) and in (**D**) the maximum number of elicitable spikes (Max N spikes). Statistical analysis was performed using one-way ANOVA followed by Bonferroni post-hoc t-test (F = 3.8, df = 3, P < 0.02 for AP; F = 10.06, df = 3, P < 0.0001, for Lat; F = 18.2, df = 3, P < 0.0001, for Ith; F = 8.1, df = 3, P < 0.0001 for N spikes). *Significantly different vs. age-matched control group (at least P < 0.05). (I) Excitability parameters of EDL muscle fibers of MLC/SOD1^G93A^ animals. Values are expressed as mean ± S.E.M. from 10–20 fibers. The excitability parameters were recorded in the absence and in the presence of 50 µM ACTZ. To note, because of the Log scale, the measure units are omitted and are: for Action Potential amplitude (AP) mV; for the threshold current amplitude (Ith) nA; for the latency of AP (Lat) msec; and for the maximum number of elicitable AP (N spikes). Statistical analysis was performed using one-way ANOVA followed by Bonferroni post-hoc t-test (F = 26.86, df = 2, P < 0.0001, for N spikes). *Significantly different vs. WT (P < 0.05) °Significantly different vs. MLC/SOD1^G93A^ (P < 0.05).

The excitability parameters were recorded in EDL muscle of SOD1^G93A^ mice and in muscle specific MLC/SOD1^G93A^ transgenic mice. Representative traces of the active parameters measured in EDL muscle of SOD1^G93A^ mice and relative WT, are shown in Fig. 6(A–D). In accord with the reduction of gCl the excitability parameters were modified (Fig. 6E–H). In particular, the Latency of action potential (Lat, the delay from the beginning of the current pulse to the onset of an action potential at threshold, in milliseconds) was increased in SOD1^G93A^ mice at 90 and at 130 days of age (+117.7 ± 29.9% and +87.7 ± 20.8%, respectively) with respect to their age-matched WT. The threshold current for the onset of the first action potential (Ith, in nanoamperes) was decreased at both age-points (−64.8 ± 5.6% and −44.4 ± 9.6%, respectively) and the maximum number of elicited spikes was increased (+55.4 ± 15% and +77.5 ± 18%, respectively). Also, we found a decrease of the amplitude (AP, in millivolts) of action potential (by −22.1 ± 6.3%) only at 130 days of age. The excitability parameters of sarcolemma have been measured also in EDL muscle fibers of MLC/SOD1^G93A^ mice and their strain-matched controls (Fig. 6I). In general, active parameters of MLC/SOD1^G93A^ mice were less affected with respect to SOD1^G93A^ ones. Indeed, only the maximum number of elicited spikes was significantly increased by +72.3 ± 11.7% with respect to WT. Importantly, the application of 50 µM ACTZ significantly restored the maximum number of spikes toward control value.

### ATP-sensitive potassium channels (KATP) activity in skeletal muscle of SOD1^G93A^ mice

**Figure 7:**
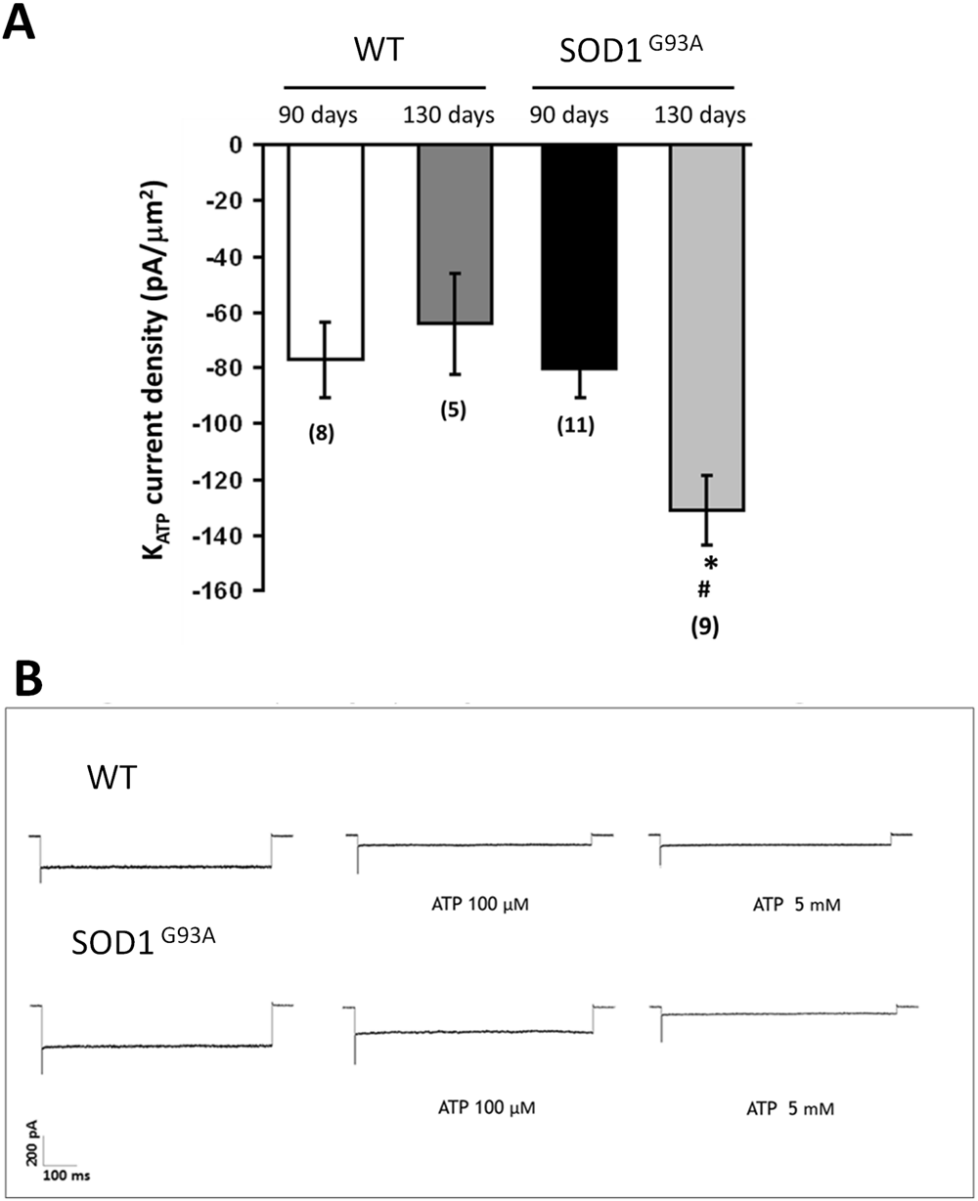
KATP channel current density from WT and SOD1^G93A^ mice measured by patch clamp technique. Each bar is the mean ± SEM from the number of patches indicated in brackets. (**A**) significant difference among groups was found by one-way ANOVA analysis (F = 7.2; P < 0.0002). Bonferroni post hoc correction for individual differences between groups is as follows: significantly different * vs WT 130 days (P < 0.002); # vs SOD1^G93A^ 90 days (P < 0.002). (**B**) Exposure of macropatches to intracellular ATP (100 µM–5 mM) induce a KATP current inhibition. The response of KATP channels to 100 µM ATP is changed in 130 days-old SOD1^G93A^ mice.

Patch-clamp experiments were performed on fast-twitch flexor digitorum brevis (FDB) muscle fibers to test whether the ubiquitous expression of SOD1^G93A^ can modify the activity of KATP channels, which are known to be altered during various physiopathological conditions^24^. The current amplitude of the KATP channel was increased in the FDB muscle of SOD1^G93A^ mice with respect to WT at 130 days (Fig. 7A). Exposure of macropatches to intracellular ATP (100µM-5mM) greatly reduced the current amplitude in control conditions, confirming that these currents flowed through KATP channels (Fig. 7B). However, KATP channels of SOD1^G93A^ mice were less sensitive to 100 µM ATP.

### Calcium Homeostasis and response to caffeine in skeletal muscle of SOD1^G93A^ and of MLC/SOD1^G93A^ mice

**Figure 8:**
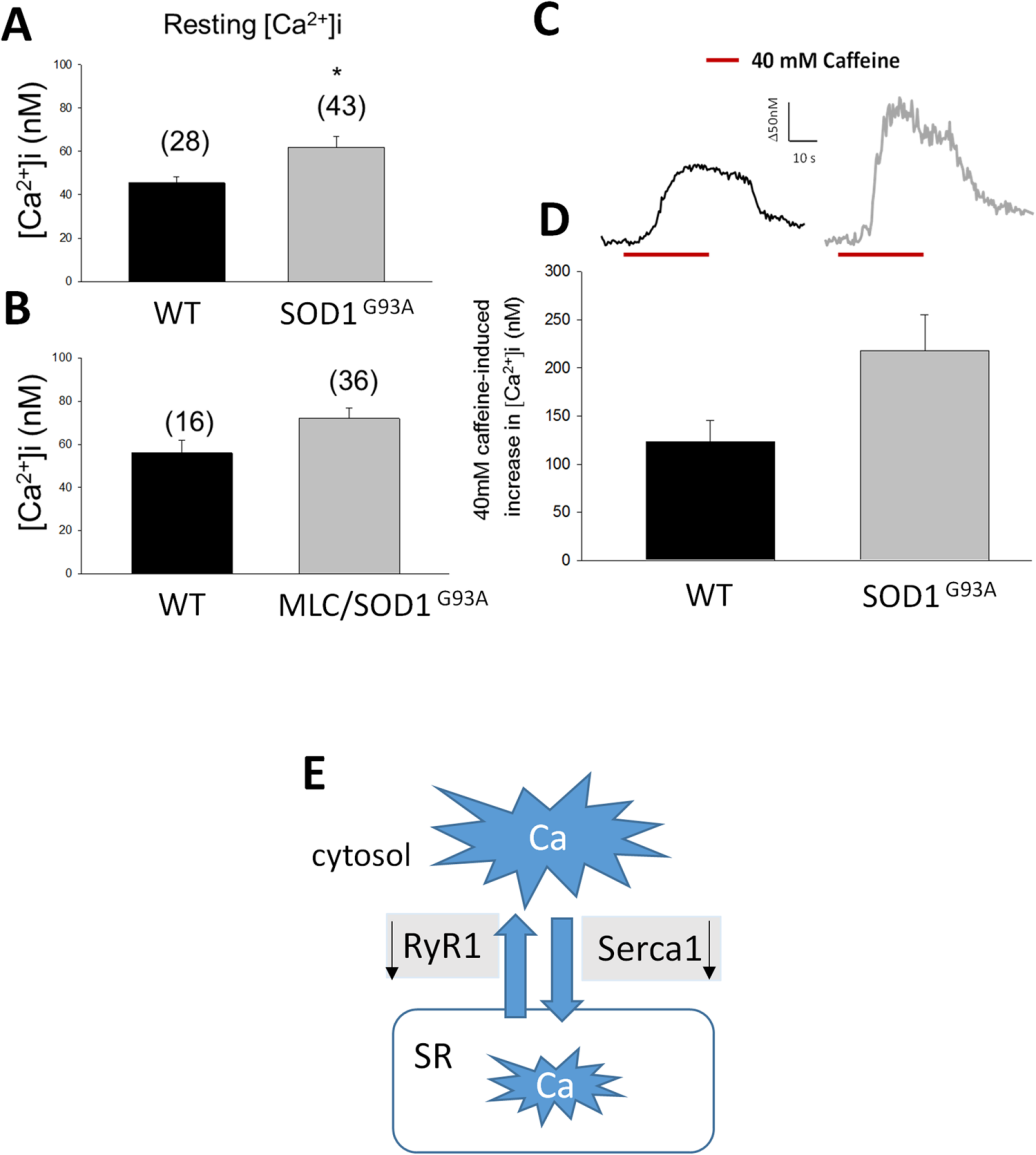
Cytosolic resting calcium (restCa) level in EDL muscle fibers measured by FURA-2 cytofluorimetric technique. RestCa level measured in (**A**) 130 days-old SOD1^G93A^ animals and in (**B**) 140 days-old MLC/SOD1^G93A^ animals. *Significantly different with respect to WT animals (P < 0.05 by Unpaired Student t-test). (**C**) Representative traces showing a 40 mM caffeine-induced increase of restCa in WT (black trace) and in SOD1^G93A^ (gray trace) mice. (**D**) Correspondent barplot constructed using the mean values ± S.E.M. obtained from 15–20 fibers. (**E**) Schematic representation of the decreased expression of RyR1 and SERCA1 mRNA (black arrows) responsible for the cytosolic calcium increase. Although the decreased expression of RyR in SOD1 ^G93A^ may indicate the reduction of Ca flux from SR to the cytosol, SERCA reduction slow down the reuptake in the SR. Slight modification in restCa level were observed in MLC/SOD1^G93A^ animals in line with the lack of changes in RyR and SERCA expression.

Using the FURA-2 cytofluorimetric technique, we measured calcium homeostasis in EDL muscle fibers of SOD1^G93A^ and MLC/SOD1^G93A^ mice in comparison to strain-matched WT (Fig. 8,B). Muscle fibers of SOD1^G93A^ mice showed a significant increase in resting cytosolic calcium (restCa) concentration with respect to WT. *In-vitro* application of 40 mM caffeine, a modulator of RyR, on EDL fibers of SOD1^G93A^ mice, induced a slight increase of restCa suggesting higher sensitivity to the compound (Fig. 8C,D). The restCa level was not significantly modified in skeletal muscle of MLC/SOD1^G93A^ mice.

## Discussion

In this study we tested for the first time the activity and expression of various skeletal muscle ion channels, which are pivotal for the function of muscle and of neighboring tissues. The analytical strategy performed allowed the identification of skeletal muscle expression signature, based both on differential gene expression and on gene correlation networks. The results show a clear-cut segregation of muscles in separated groups: SOD1^G93A^ and MLC/SOD1^G93A^ versus their strain-matched WT. The PCA analysis of gene expression data showed no separation between 90 days-old and 130 days-old animals suggesting that skeletal muscle is severely involved in ALS pathogenesis already before the appearance of clear symptoms. In ALS patients, muscle denervation and abnormal reinnervation are pathological hallmarks of the disease. Similarly, an up-regulation of denervation markers (Nav1.5, AChR)^8,25,26^ was found in SOD1^G93A^ muscles at both time points, together with an increase of myogenin^27–29^ that indicates muscle reinnervation process^27^. In addition, inhibition of Mstn, a negative regulator of muscle mass, suggests an attempt of the muscle to counteract atrophy and promote regeneration. Interestingly, Mstn expression was decreased also in MLC/SOD1^G93A^ mice, the animal model expressing the SOD1 mutant gene selectively in skeletal muscle^4^, again suggesting the early involvement of skeletal muscle in the pathogenesis of ALS. A fast-to-slow phenotype transition was observed in SOD1^G93A^ mice, likely as an attempt to convert muscle fibers into a more resistant slow type, less affected by the pathology. In skeletal muscles of these mice, the expression of ClC-1 channel and the related resting gCl were significantly decreased with respect to WT, at 90 and 130 days of life. This is in accord with the fast-to-slow phenotype transition, since ClC-1 expression and resting gCl in slow-twitch muscles are lower than in the fast ones^30^. The ClC-1 protein expression and resting gCl were significantly reduced also in muscles of MLC/SOD1^G93A^ mice, likely through a mechanism involving Reactive Oxygen Species (ROS) production. An in-depth analysis revealed that the reduction of gCl in both models also involved PKC-theta up-regulation, which phosphorylates and inhibits the ClC-1 channel^13,31^. According to the reduction of gCl, sarcolemma excitability was increased, with a significant increase of the maximum number of spikes. The situation was more complex in SOD1^G93A^ mice, since other channels were involved. In these mice a decreased amplitude of the action potential observed in SOD1^G93A^ mice can be in part explained by the down-regulation of Nav1.4 sodium channel transcript. The increase in resting gK is likely sustained by the increased KATP current in SOD1^G93A^ mice, although we cannot exclude the contribution of other K channels. The KATP current is myoprotective and may be activated to dampen excitability. However, the mRNA expression of the pore subunit Kir6.2 and the auxiliary subunit SUR2A was decreased, but it is possible that the increased expression of SUR1, more resistant to oxidative stress, may account for the increased KATP current. Moreover, the modification of the channel and/or the phenotype shift^32^ may explain the ATP resistance in these mice. All these modifications were not observed in the MLC/SOD1^G93A^ mice. Likewise, calcium channels were strongly involved in the modification of skeletal muscle function in SOD1^G93A^ mice. The restCa was significantly increased in the EDL muscles according to the fast-to-slow phenotype transition. Despite a reduction of RyR mRNA expression, a strong decrease of SERCA1, responsible for SR calcium reuptake, may contribute to the increase of restCa. Also a depletion of ATP may reduce SERCA1 activity. Interestingly, the increase of SERCA2 expression may represent an attempt to compensate for the loss of SERCA1^33–35^. In MLC/SOD1^G93A^ model, the not significant increase of restCa and the unchanged expression of RyR and SERCA1, both suggest a minor involvement in this strain. Other studies^36^ have shown an impairment of EC coupling in FDB fast-twitch fibers of both models. All the modifications observed in skeletal muscle of SOD1^G93A^ animals seems to be associated to denervation and phenotype transition. In contrast, no sign of morphological denervation was found in MLC/SOD1^G93A^ mice, at the examined age, where motor neurons are not affected. However muscle restricted expression of SOD1^G93A^ displays alteration in NMJ^4^. MLC/SOD1^G93A^ muscles were characterized by a minor number of transcriptional modifications, and the identified most discriminant genes involved in the onset of damage were PKC-theta, that control muscle ClC-1 activity, AMPAR2, the ionotropic glutamate receptor involved in synaptic plasticity, and irisin, a recently-discovered hormone secreted by skeletal muscle and able to communicate in a paracrine and endocrine manner with nervous system^37^. The functional significance of an increase of AMPAR2 may be the facilitation of glutamate-induced prolonged depolarization and modulation of messengers (e.g. NO) able to activate retrograde potentiation in pre-synaptic compartments^38,39^. The consequent muscle hyperexcitability may rapidly exacerbate the damage at neuronal tissue^9^, already compromised by the increase of firing rates, ionic imbalance and sustained depolarization that decreases ATP level^21,35^. The results indicate that muscle hyperexcitability is due to ClC-1 expression reduction and increased activity of PKC-theta with consequent gCl reduction in MLC/SOD1^G93A^ mice. The increased activity of PKC was confirmed by the *in-vitro* effects of chelerythrine, a well known inhibitor of PKC, which was able to increase the resting gCl^23^. Also, AMPAR2 activation can be modulated by PKC-induced increased phosphorylation^40^. Our hypothesis is that selective accumulation of mutant SOD1 in muscle and consequent oxidative stress may affect PKC, ion channel function, and neuromuscular communication^41^. In this situation, a “feedback-loop” could be triggered, which may explain why motor neuron death begins with an initial distal hyperexcitability^42–44^. Accordingly, muscle-specific expression of local Igf-1 (mIgf-1) was shown to stabilize NMJ, reduce inflammation in the spinal cord, and enhance motor neuronal survival in SOD1^G93A^ mice, thereby delaying the onset and progression of the disease^5,8^. We already demonstrated that Igf-1 signaling is important to maintain a normal phosphatase activity that counterbalances the inhibitory activity of PKC on ClC-1^45,46^. Thus, it is likely that the decrease of phosphatase activity together with PKC activation strongly contribute to gCl reduction and hyperexcitability in ALS. Since gCl is strongly reduced during aging in skeletal muscle of rodents^12^, it is possible that more severe alterations in MLC/SOD1^G93A^ mice may occur at advanced age, as already observed^5^. Accordingly, preliminary data showed a more severe reduction of gCl in 10-months-old MLC/SOD1^G93A^ mice (Supplementary Fig. S6). All these findings identify ACTZ as a potential drug for ALS therapy. As already demonstrated^11,14,47,48^, this carbonic anhydrase inhibitor, was able to increase ClC-1 channel activity likely through modification of internal pH. The increased expression of carbonic anhydrase in motor neurons of ALS patients also supports ACTZ therapeutic proposal^49^. Notwithstanding the encouraging results in the restoration of gCl and attenuation of muscle hyperexcitability in MLC/SOD1^G93A^ mice, additional preclinical studies are required to better assess the long-term effects of ACTZ. Irisin is another discriminant gene, found to be down-regulated in MLC/SOD1^G93A^ mice. Irisin is a myokine secreted by both skeletal muscle and brain. Physical activity modifies plasma concentration of this hormone known to stimulate the synthesis of brain-derived neurotrophic factor (BDNF) in the nervous system^50^. Thus, irisin associates skeletal muscle activity to that of nervous system in a centripetal manner, being able to cross the blood–brain barrier^51^. Irisin gene expression was modified not only in the MLC/SOD1^G93A^ mice, but also in SOD1^G93A^ mice, suggesting that its modification is an early and long-lasting event in the pathogenesis of ALS. Based on these results, irisin may represent a critical link between muscle and CNS in ALS and a likely pharmacological target. Thus, a combined therapy with irisin may be also considered to restore muscle-nerve communication. ACTZ, which increases ClC-1 activity, and riluzole, which inhibit Na^+^ channels activity^14^, might act synergically to restore muscle excitability. Our results strengthen the evidence for the role of skeletal muscle in ALS pathogenesis and pave the way for the development of new therapeutic options to hamper the clinical effects of the disease.

## Material and Methods

### Animal care

In this study two different animal models of ALS were evaluated. Male SOD1^G93A^ transgenic mice ubiquitously expressing the human mutant SOD1 G93A allele containing the Gly93RAla (G93A) substitution were used at two time points (90 and 130 days-old) based on the criteria for disease progression^52^ as clinical onset (mice selected showed no overt sign of pathology) and early symptomatic (mice were selected at abnormal gait and before paralysis). Male MLC/SOD1^G93A^ mice (140 days-old) overexpressing the mutant SOD1G93A transgene under the transcriptional control of the Myosin Light Chain (MLC) muscle specific promoter, so that its expression is selectively restricted in skeletal muscle^4^. Transgenic animals were identified by PCR amplification of DNA extracted from tail tissue^44^. Age-matched C57BL6J and FVB/NJ mice (Jackson Laboratories, Bar Harbor, ME, USA) were used as WT control strain, respectively. The animals were housed in a temperature-controlled (22 °C) room with a 12:12 h light-dark cycle. Before muscle dissection animals were deeply anesthetized with ketamine (100 mg/kg ip)/xylazine (16 mg/kg ip). The experiments were approved by the ethics committee of the University of Bari and by the Veterinary authority of the Italian Ministry of Health (D.M. n.126/2009, Authorization 386/2017-PR) and were performed in accordance with the Italian Guidelines for the Use and Care of Laboratory Animals (D. Lgs 2014 n26), which conforms with the European Communities Council Directive of 24 November 1986 (86/609/EEC) and Guidelines from Directive 2010/63/EU of the European Parliament on the protection of animals used for scientific purposes.

### Isolation of total RNA, reverse transcription and real-time quantitative polymerase chain reaction (PCR) analysis

Immediately after surgery, TA muscles were snap frozen in liquid nitrogen and stored at −80 °C until isolation of total RNA, reverse transcription and real-time PCR analysis, as detailed in Camerino *et al*.^16^. Genes were analysed by the use of TaqMan Hydrolysis primer and probe gene expression assays that are produced by Life-Technologies with the following assay IDs: PKC theta (encoded by *Prkcq* gene) assay ID: Mm00436796_m1; PKC alpha (encoded by *Prkca* gene) assay ID: Mm00440858_m1; Kir 6.2 (encoded by *Kcnj11* gene) assay ID: Mm00440050_g1; Sur1 (encoded by *Abcc8* gene) assay ID: mm008003450_m1; Ryr1 (encoded by *Ryr1* gene) assay ID: Mm01175211_m1; Nav1.4 (encoded by *Scn4a* gene) assay ID: Mm01258366_m1; Nav1.5 (encoded by *Scn5a* gene) assay ID: Mm01342505_m1; Serca1 (encoded by *Atp2a1* gene) assay ID: Mm01275320_m1; Serca2 (encoded by *Atp2a2* gene) assay ID: Mm01201431_m1; CN (Calcineurin, encoded by *Ppp3ca* gene) assay ID: Mm01317678_m1; Irisin (encoded by *Fndc5* gene) assay ID: Mm01181543_m1; Nmdar1 (encoded by *Grin1* gene) assay ID: Mm00433790_m1; Ampar2 (encoded by *Gria2* gene) assay ID: Mm00442822_m1; Notch1 (encoded by *Notch1* gene) assay ID: Mm00435249_m1; TauT (encoded by *Slc6a6* gene) assay ID: Mm01264789_m1; Murf-1 (encoded by *Trim63* gene) assay ID: Mm01185221_m1; Mhc1, myosin heavy chain 1 (encoded by *Myh7* gene) assay ID: Mm00600555_m1; Mhc2a, myosin heavy chain 2a (encoded by *Myh2* gene) assay ID: Mm00454982_m1; Mhc2b, myosin heavy chain 2b (encoded by *Myh4* gene) assay ID: Mm01332541_m1; Mhc2x, myosin heavy chain 2 × (encoded by *Myh1* gene) assay ID: Mm01332489_m1; Ampk (encoded by *Prkaa1* gene) assay ID: Mm0164789_m1; Acetylcholine Receptor, Nicotinic, Alpha 1, Achr1 (encoded by *Chrna1* gene) assay ID: Mm00431629_m1; Ngf (encoded by *Ngf* gene) assay ID: mm00443039_m1; Myostatin (encoded by *Mstn* gene) assay ID: Mm01254559_m1; Beta-2-Microglobulin, B2m (encoded by *B2m* gene) assay ID: Mm00437762_m1. For β-actin (*Actb* gene) we designed primer For: 5′-CCAGATCATGTTTGAG ACCTTCAA-3, primer Rev: 5′-CATACAGGGACAGCACAGCCT-3, probe: VIC-ACC CCA GCC ATG TAC GTAMGB. For chloride channel ClC-1 (*Clcn1* gene) we designed primer For: 5′-TCATGCTCGGTGTCCGAAA-3′, primer Rev: 5′-CAGGCGGTGCTTAGCAAGA-3′, probe: 6-FAM-ATTGGCTGAGACACTTGT-MGB. For BK channel (*Kcnma1* gene) we designed primer For: 5′-GATATCCGCCCAGACACTGAC-3′, primer Rev: 5′-AGTATATTACGAGGGGACCAA-3′, probe: VIC-CAGAGTCCTGGTTGTGTTA-MGB; For Sur2a (*Abcc9* splicing A gene) we designed primer For: 5′-ACATGGCCACGGAAAACATT-3′, primer Rev: 5′-ACTCCACTAAAATACCCTCAGAAAAGA-3′, probe: FAM-CCATAGCTCACCGTGTCT-MGBQ; For Sur2b (*Abcc9* splicing B gene) we designed primer For: 5′-CGGATCGACGGTCGTA-3′, primer Rev:5′-TAACCAGGTCTGCAGTCAGAATG-3′, probe: FAM-CATAGCTCATCGGGTTC-MGBQ^53^. The mRNA expression of the genes was normalized to the best housekeeping gene: *Actb* selected from *B2m* and *Actb* by Normfinder software^16^. For genes that are poorly expressed, such as *Ampar2*, *Nmdar1*, *Notch1*, *Ngf*, *Mstn* pre-amplification by TaqMan PreAmp Master Mix (Life Technologies C.N. 4391128) was made before the real-time experiments.

### Western Blot analysis

ClC1 protein was isolated from TA muscle of MLC/SOD1^G93A^ and processed as described in Camerino *et al*.^16^. Primary rabbit anti-ClC1 antibody (MyBiosource) was diluted 1:200 with TBS containing 5% non-fat dry milk overnight. Membranes were incubated for 1 h with secondary antibody labeled with peroxidase (1:5000 anti rabbit IgG, Sigma-Aldrich), developed with a chemiluminescent substrate (Clarity Western ECL Substrate, Bio-Rad) and visualized on a Chemidoc imaging system (Bio-Rad). Densitometric analysis of each experimental band was performed using Image Lab software (Bio-Rad). The software allows the chemiluminescence detection of each experimental protein band to obtain the absolute signal intensity. The density volume was automatically adjusted by subtracting the local background. For each sample, the relative intensity was calculated by normalizing the intensity of β–actin protein band (diluted 1:300, rabbit Anti Actin, Santa Cruz Biotechnology) as reference standard.

### Immunofluorescence

TA muscles, covered with tissue-tek O.C.T. (Bio-Optica), were frozen in isopentane cooled in liquid nitrogen in a slightly stretched position and stored at −80 °C. Serial cross sections (8-μm thick) were cut in a cryostat microtome set a −20 °C (Thermo Scientific) and incubated with primary antibody against rabbit ClC-1 diluted 1:100 (MyBiosource) in PBS-gelatin for 1 h. After washing with PBS-gelatin, the sections were incubated with 488-donkey anti rabbit IgG (Invitrogen), diluted 1:1000 in PBS-gelatin for 1 h, then washed with PBS-NaCl (300 mM). Sections were examined using Olympus CX41 microscope^53^.

### Recordings of Resting Chloride and Potassium Conductances and Excitability Parameters in skeletal muscle of SOD1^G93A^ and in muscle specific MLC/SOD1^G93A^ transgenic mice measured in current clamp mode by the Two-Intracellular Microelectrodes Technique

Extensor Digitorum Longus (EDL) and Soleus (Sol) muscles were fixed by tendons to a glass rod immersed in normal (NP) or chloride-free physiological solution maintained at 30 °C and perfused with 95% O2/5% CO2^23^. The NP solution contained (in mM): NaCl 148, KCl 4.5, CaCl2 2.0, MgCl2 1.0, NaHCO3 12.0, NaH2PO4 0.44, glucose 5.5, and pH 7.2. The chloride-free solution was prepared by equimolar substitution of methylsulfate salts for NaCl and KCl and nitrate salts for CaCl2 and MgCl2. The cable parameters of myofiber sarcolemma were determined from the electrotonic potentials elicited by square wave hyperpolarizing current pulse of 100-ms duration, using two intracellular microelectrodes in current-clamp mode, as previously described^30,31,54^. The membrane conductance is calculated from the values of input resistance, space constants and time constant and assuming a myoplasmic resistivity of 125 Ω cm. The mean chloride conductance (gCl) is calculated as the mean total membrane conductance (gm) measured in NP solution minus the mean potassium conductance gK measured in chloride-free solution. Sarcolemma excitability parameters were determined by applying 100ms-long depolarizing current pulses of increasing amplitude to elicit first a single action potential (AP) then a train with the maximal number of APs. The membrane potential was held at −80 mV between test pulses. A single and a train of action potentials were generated by increasing current intensity in the same fiber and the excitability characteristics were measured (amplitude of action potential, threshold current, latency of action potential and N spikes)^31,55^.

### Measurement of KATP channel activity in skeletal muscle of SOD1^G93A^ and in muscle specific MLC/SOD1^G93A^ transgenic mice using whole-cell patch-clamp technique

Patch-clamp experiments were performed on freshly enzymatically dissociated FDB muscle fibers in inside-out configurations using the standard patch-clamp technique. The ATP-sensitive potassium (KATP) channel currents were recorded immediately after excision during voltage steps from 0 to −60 mV with 150 mM KCl on both sides of the membrane patches in the absence (control) or the presence of ATP in the muscle bath^24^. The currents were recorded at a 1-kHz sampling rate (filter = 0.2 kHz) using an Axopatch-1D amplifier equipped with a CV-4 head stage (Axon Instruments, Union City, CA). Macropatches having an average pipette area of 11.3 ± 1 μm^2^ were used to measure the mean KATP currents, which were calculated by subtracting the baseline level from the open-channel level of each current trace and then digitally averaging all generated files using CLAMPFIT (Axon Instruments). The baseline level for the KATP current was measured in the presence of internal ATP (5 × 10−3 M). Current amplitude was measured using CLAMPFIT.

### Fluorescence Measurements of Resting Intracellular Ca^2+^ Concentration in skeletal muscle of SOD1^G93A^ and in muscle specific MLC/SOD1^G93A^ transgenic mice using FURA-2 imaging technique

Fluorescence measurements were performed on small bundles of five to ten fibers lengthwise dissected from mice EDL muscles, as described elsewhere^56^. The muscle fibers were incubated with the fluorescent calcium probe FURA-2 for 45–60 min at 22 °C in physiological solution containing 5 µM of the acetoxymethyl ester (AM) form of the dye mixed to 10% (v/v) Pluronic F-127 (Molecular Probes, Leiden, The Netherlands). A QuantiCell 900 integrated imaging system (VisiTech International Ltd) was used to acquire pairs of background-subtracted images of the FURA-2 fluorescence emission (510 nm) excited at 340 nm and 380 nm. The equation used to transform fluorescence ratio in [Ca^2+^]i values was [Ca^2+^]i = (R–Rmin)/(Rmax–R) •K_D_•β, where R is the ratio of fluorescence excited at 340 nm to that excited at 380 nm; K_D_ = 145 nM; β, Rmin and Rmax were determined *in situ* in ionomycin- permeabilized muscle fibers. The cytosolic calcium response of muscle fibers to *in vitro* application of 40 mM caffeine was measured to evaluate calcium release from the sarcoplasmic reticulum.

### Statistical analysis and Multivariate Statistical analysis: PCA and LDA algorithms

All data are expressed as the mean ± SEM. Statistical analysis for direct comparison between two groups of data means was performed using unpaired Student’s t-test, while multiple statistical comparison between groups was performed using ANOVA test, with Bonferroni’s t test post hoc correction, allowing a better evaluation of intra- and inter-group variability (using Prism 7.0 Software, GraphPad). For the analysis of correlations between two parameters, Python 3.6, Scipy and Seaborn packages have been used and Pearson correlation coefficient and the 2-tailed p-value for testing non-correlation have been calculated. Multivariate analysis of gene expression dataset has been performed following the PCA-LDA analysis^57,58^. For each animal 27 genes have been analyzed as normalized values ratio with house-keeping gene β-Actin (HK: Actb; target genes: ClC1, PKC Theta, PKC Alpha, Ryr1, Serca1, Serca2, CN, Bk, Kir6.2, Sur1, Surb2a, Sur2b, Nav1.4, Nmdar1, Ampar2, Murf1, Irisin, Notch1, TauT, Ampk, Ngf, Mstn, Achr1, Mhc2b, Mhc2x, Mhc2a, Mhc1). The starting data-frame consisted of a matrix with 30 rows (animals) and 27 columns (gene expression levels) with a total of 810 points. Because PCA and LDA algorithms suffer of the presence of big outliers in the dataset, first step of the pipeline has been their handling. So, the two biggest outliers onto genes that represents the major part of the variance in the system of data (ESD method, p < 0.001) have been removed and the resulting empty cells of table have been filled with the mean values of the mRNA’s level of the groups to which they belong. The second step has been to standardize the gene expression dataset scaling the mean to 0 and the variance to unitary scale, this is done for each column. After that a PCA object has been trained by the use of the Scikit-learn class sklearn.decomposition.PCA setting the number of principal component computed up to 27 (the minor dimension of the dataset) and the svd-solver parameter to ‘full’, because the dataset has less than 500 × 500 dimension. In this way, the object PCA has been trained, taking as input, the consolidated and standardized dataset, computing than a linear dimensionality reduction using the Singular Value Decomposition solver. The subsequent step in the PCA-LDA pipeline was to perform 3 Fisher Linear Discriminant Analysis (LDA) of PC scores. The class sklearn.discriminant_analysis has been used to build a prototype classifier with a linear decision boundary using the Bayes’ rule, starting from a matrix n x m and a vector containing group identifiers. The 3 LDA further reduced dataset’s dimension starting the first three PC scores, to an single Linear Discriminant (LD) dimension, in a supervised manner, maximizing the variance between and minimizing the variance within classes. From each LDA a 1 × 3 vector with the LD coefficients of the 3 PC have bene calculated. In the first LDA we obliged the classifier to find the LD that better separate MLC/SOD1^G93A^ mice from WT. In the second LDA the LD that better separate SOD1^G93A^ mice from the WT and in the third LDA the LD that better segregate MLC/SOD1^G93A^ mice from the SOD1^G93A^ ones. Finally, 3 vectors of LDA coefficients and a matrix containing the PCA loading weights of each genes have been obtained. Thus, the three discriminant lists of genes that separate the classes have been obtained multiplying the PCA loadings matrix with each transposed vector of LDA coefficients. Each multiplication gave as result, a vector of 27 PCA-LDA discriminant weights of genes analyzed. The absolute values of each element in these lists have been ordered in growing manner so the most important discriminant genes appeared at the bottom of the three lists of discriminant genes.

## Supplementary information

Supplementary Material. Elucidating the Contribution of Skeletal Muscle Ion Channels to Amyotrophic Lateral Sclerosis in search of new therapeutic options
