# Supplementary material for: Elucidating the Contribution of Skeletal Muscle Ion Channels to Amyotrophic Lateral Sclerosis in search of new therapeutic options
Source: Sci Rep. 2019 Feb 28;9:3185. doi: 10.1038/s41598-019-39676-3 (PMC6395744; doi:10.1038/s41598-019-39676-3)
Supplement: Supplementary file 1 — Supplementary Material. Elucidating the Contribution of Skeletal Muscle Ion Channels to Amyotrophic Lateral Sclerosis in search of new therapeutic options [file 41598_2019_39676_MOESM1_ESM.pdf]

Supplementary Material

Elucidating the Contribution of Skeletal Muscle Ion Channels to Amyotrophic Lateral Sclerosis in search of new therapeutic options

Giulia Maria Camerino[1a], Adriano Fonzino[1a], Elena Conte[1], Michela De Bellis[1], Antonietta Mele[1], Antonella Liantonio[1], Domenico Tricarico[1], Nancy Tarantino[1], Gabriella Dobrowolny[2], Antonio Musarò[2], Jean-Francois Desaphy[3], Annamaria De Luca[1] & Sabata Pierno[1*]

[1]Department of Pharmacy-Drug Sciences, University of Bari Aldo Moro, 70125 Bari, Italy

[2]DAHFMO-Unit of Histology and Medical Embryology, Sapienza University of Rome, 00161 Laboratory affiliated to Istituto Pasteur Italia – Fondazione Cenci Bolognetti, Italy and Center for Life Nano Science at Sapienza, Istituto Italiano di Tecnologia, 00161 Rome, Italy

[3]Department of Biomedical Sciences and Human Oncology, University of Bari Aldo Moro, Polyclinic, 70124 Bari, Italy.

[a]G.M.C. and [a]A.F. contributed equally to this work as first authors

*Corresponding author:

Sabata Pierno, Section of Pharmacology, Department of Pharmacy-Drug Sciences, University of Bari Aldo Moro, Via Orabona 4, 70125, Bari, Italy, Tel: 0039-080-5442802; E-mail: sabata.pierno@uniba.it

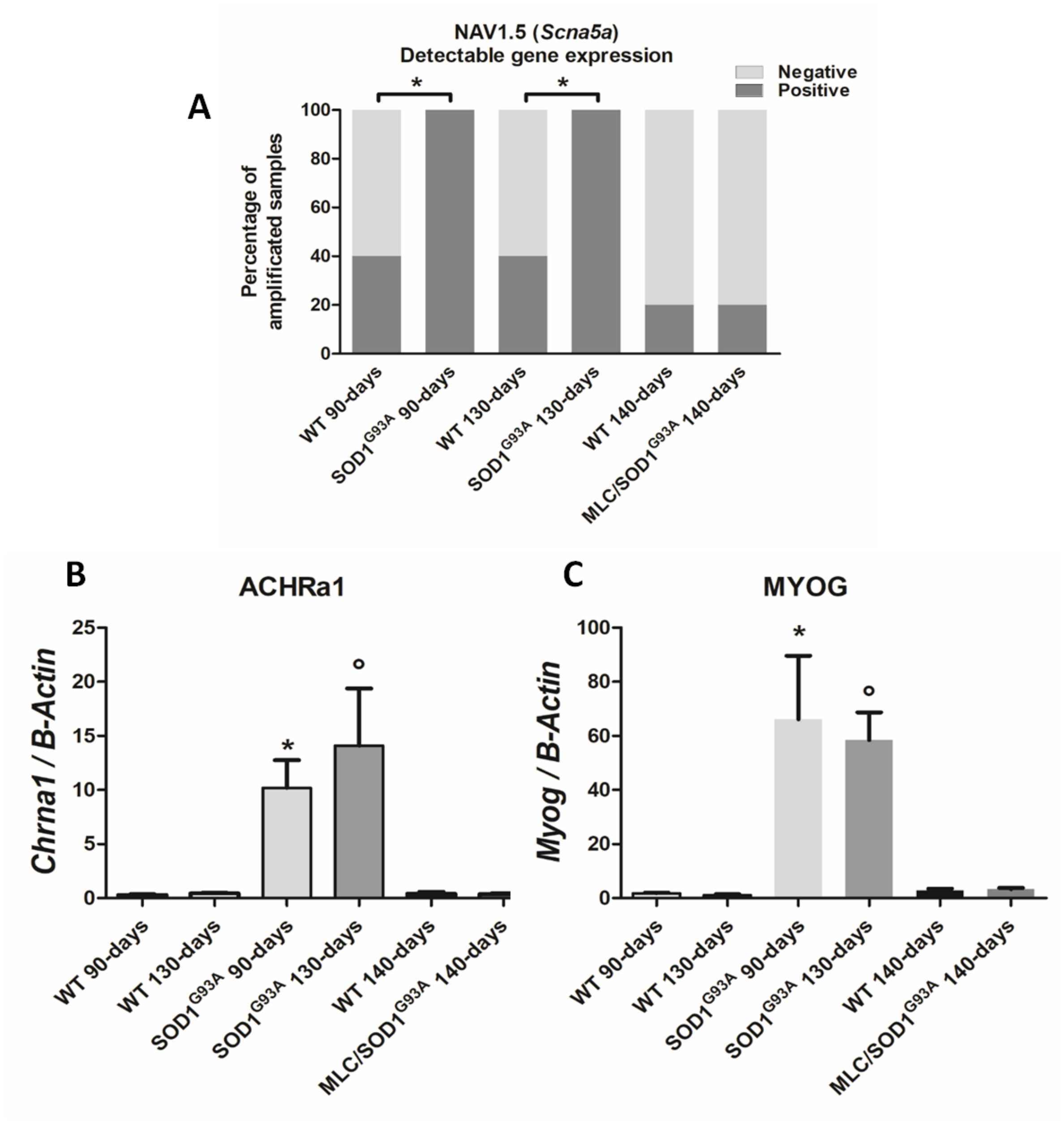

Supplementary Fig. S1. Gene expression of denervation markers in skeletal muscle of SOD1^{G93A} and in muscle specific MLC/SOD1^{G93A} transgenic mice. (A) Percentage of muscle samples with detectable gene expression level of Nav1.5 mRNA. Nav1.5 is an isoform of the voltage gated Na+ channel, expressed in embryonal and in denervated adult muscle. It was strongly detectable, with positive amplifications in all samples of SOD1^{G93A} at both ages (90 and 130 days of age) with respect to wild-type (WT), in which it was absent. Each bar represents, in the corresponding experimental group, the percentage of Nav1.5 positive (dark gray) and negative (light gray) samples with detectable mRNA level. *Significantly different with respect to WT ($p<0.05$, by two-tailed Chi-square test). (B) Gene expression level of ACh Receptor (AChRa1) in skeletal muscle of WT and transgenic mice (SOD1^{G93A} and MLC/SOD1^{G93A}). AChRa1 up-regulation is typically observed in denervated muscle (Dobrowolny et al., 2005), together with myogenin, involved in the expression, stabilization and clustering of AChR. Values are expressed as AChRa1 /β-Actin gene expression level ratio as described in the Materials and Methods section. *Significantly different vs. 90 days-old WT; °vs 130 days-old WT ($p<0.05$ or less, by two-tailed Unpaired Student t-test). (C) Gene expression of the transcription factor Myogenin (MYOG) in the same samples. *Significantly different with respect to the age-matched controls ($p<0.05$ or less, by two-tailed Unpaired Student t-test). Five samples for each experimental group were analyzed. No modifications were found in MLC/SOD1^{G93A} mice.

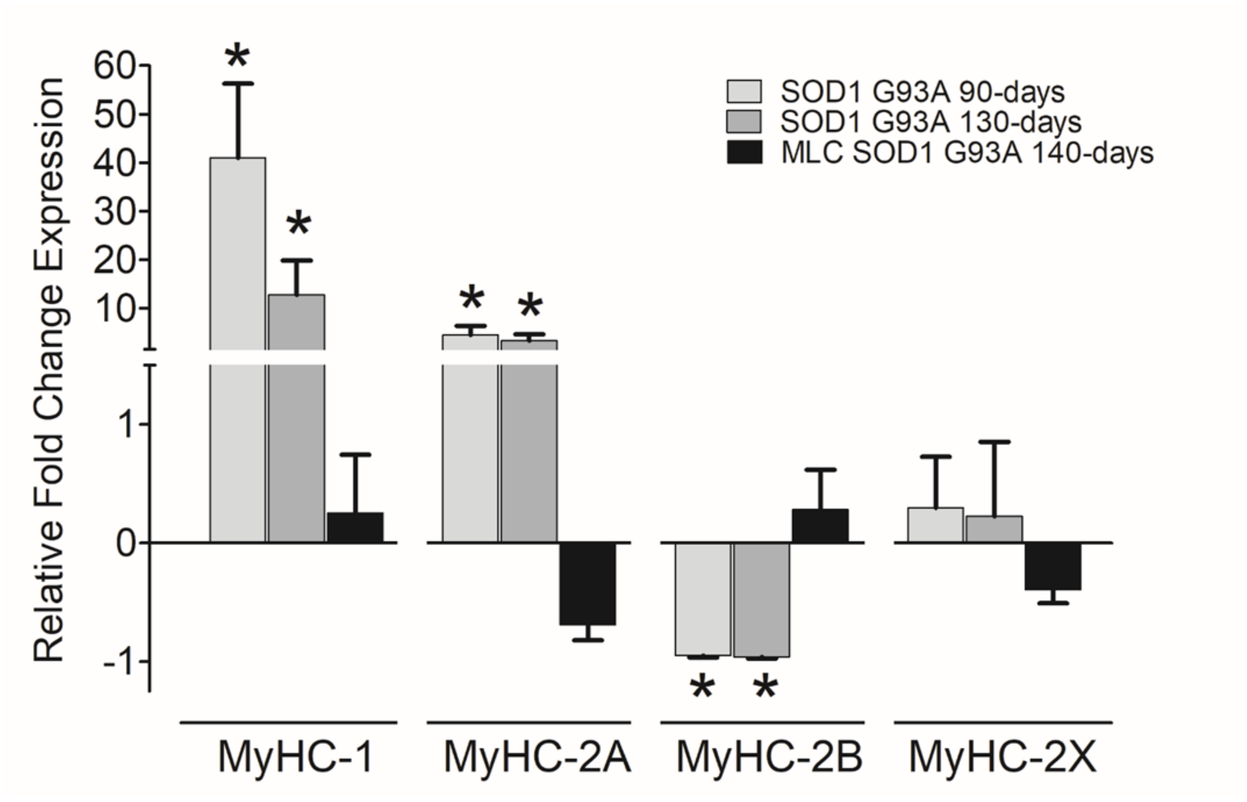

Supplementary Fig. S2. Gene expression of Myosin Heavy Chain isoforms in skeletal muscle of SOD1^{G93A} and in muscle specific MLC/SOD1^{G93A} transgenic mice. It is known that muscle atrophy and denervation, are factors that could lead to alterations in Myosin Heavy Chains (MyHC) composition in skeletal muscle fibers and phenotype transition (Raffaello et al., 2006; Desaphy et al., 2010). For this reason, we explored the mRNA expression levels of the different MyHC isoforms (MyHC-1, MyHC-2A, MyHC-2B, MyHC-2X) in Tibialis Anterior (TA) muscles of 90 days-old (light gray bars), 130 days-old (dark grey bars) SOD1^{G93A} and MLC/SOD1^{G93A} (black bars) mice. The mRNA level of the slow-type isoform MyHC-1 and MyHC-2A was significantly up-regulated in SOD1^{G93A} animals, with respect to their controls at 90 and 130 days-of-age. In contrast, mRNA level of MyHC-2B isoform, was significantly down-regulated in SOD1^{G93A} genotype, at both ages, indicating a fast-to-slow phenotype transition. The transcript levels of the various MyHC isoforms in TA muscles were not modified in MLC/SOD1^{G93A} mice. Histograms show Relative Fold Change (FC) versus strain- and age-matched controls, of transcript levels performed by Real-Time PCR, for different isotypes of MyHC genes normalized by the β-Actin gene, in the 6 experimental groups. Each bar represents the FC mean ± S.E.M. *Significantly different versus strain- and age-matched control group ($p<0.05$ by Unpaired Student t-test). Five samples for each experimental group were analyzed.

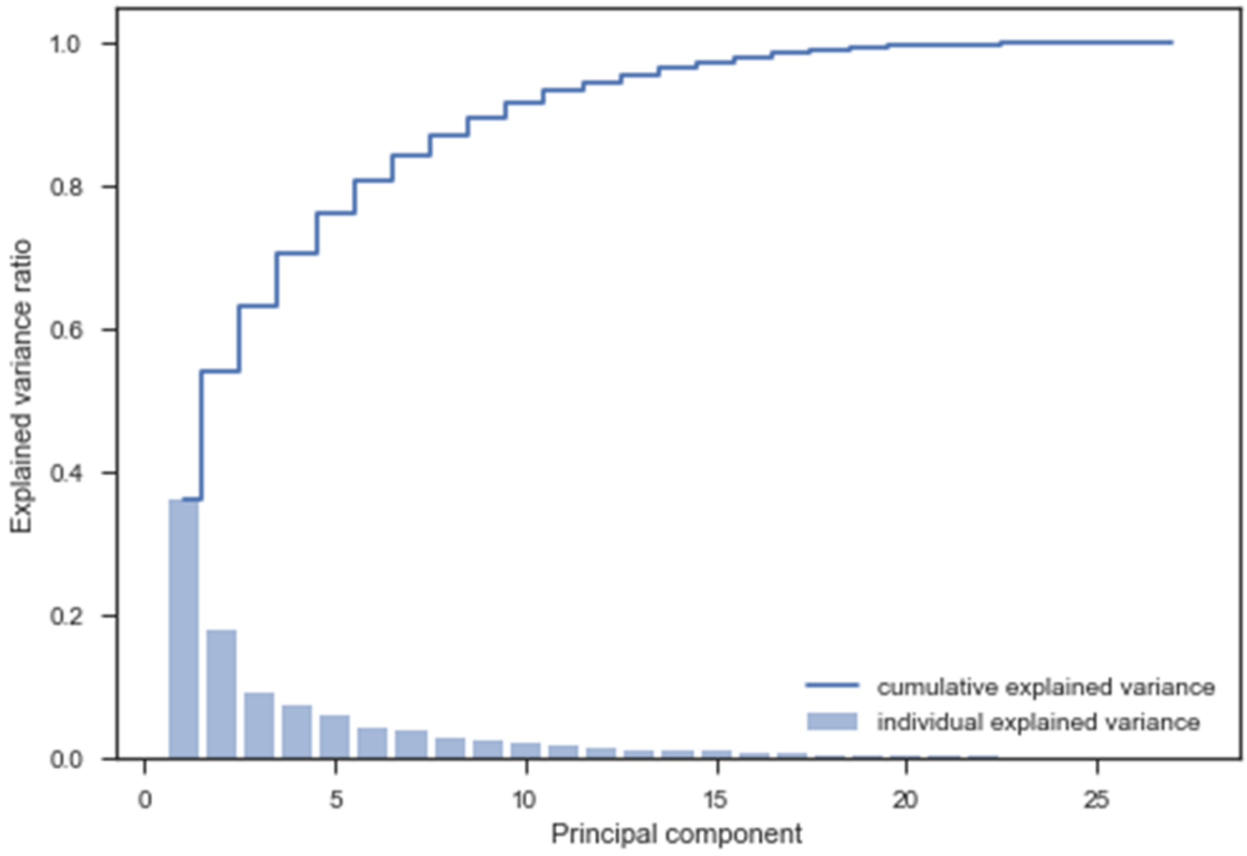

Supplementary Fig. S3. Bar Plot showing the explained variance ratio expressed by each Principal Component (PC). Bars represent the percentage of total explained variance ratio sustained by the corresponding PC. The elbow line above the bars shows the reached cumulative Explained Variance Ratio.

	PC1	PC2	PC3	PC4	PC5	PC6	PC7	PC8	PC9	PC10	PC11	PC12	PC13	PC14	PC15	PC16	PC17	PC18	PC19	PC20	PC21	PC22	PC23	PC24	PC25	PC26	PC27
**explained variance ratio**	0,361	0,178	0,092	0,073	0,058	0,043	0,037	0,027	0,025	0,021	0,017	0,012	0,011	0,009	0,009	0,007	0,006	0,004	0,004	0,002	0,002	0,001	0,001	0,001	0,000	0,000	0,000
**cumulative variance ratio**	0,361	0,539	0,631	0,705	0,762	0,805	0,842	0,869	0,895	0,916	0,932	0,945	0,956	0,964	0,973	0,980	0,985	0,990	0,993	0,995	0,997	0,998	0,999	1,000	1,000	1,000	1,000

Supplementary Table S1. Actual values of the Explained Variance Ratio and Cumulative Variance Ratio for each PC.

Eigenvectors	PC1	PC2	PC3	PC4	PC5	PC6	PC7	PC8	PC9	PC10	PC11	PC12	PC13	PC14	PC15	PC16	PC17	PC18	PC19	PC20	PC21	PC22	PC23	PC24	PC25	PC26	PC27
**Sur1**	-0,191	0,201	0,002	0,343	-0,020	0,182	-0,147	0,042	-0,316	0,010	0,161	0,158	-0,195	-0,555	-0,226	0,096	0,073	0,097	0,136	-0,128	0,128	0,205	-0,217	0,009	0,118	-0,138	0,054
**Bk**	0,256	0,081	0,081	-0,209	-0,097	-0,302	0,179	0,041	0,006	0,157	0,251	0,194	-0,118	0,115	-0,104	0,418	0,050	0,237	0,211	0,096	0,229	0,097	-0,174	0,288	-0,102	0,114	-0,306
**Pkc alpha**	-0,070	0,281	-0,013	-0,093	-0,250	0,231	0,278	-0,564	-0,049	0,379	-0,133	-0,255	0,056	-0,066	0,047	-0,252	-0,036	-0,010	0,136	0,052	0,124	0,028	-0,142	0,029	-0,152	0,055	-0,090
**Kir6.2**	0,239	0,212	-0,041	0,010	0,074	-0,318	-0,107	-0,063	-0,011	-0,137	-0,511	-0,035	0,046	-0,266	0,024	0,173	-0,039	0,258	-0,001	0,114	-0,186	-0,118	-0,296	-0,251	-0,226	0,162	0,178
**Pkc theta**	0,087	0,313	0,356	0,008	-0,105	-0,003	-0,062	0,077	0,189	0,298	0,188	-0,055	-0,218	0,116	-0,178	0,059	-0,288	-0,016	0,046	-0,084	-0,124	-0,267	0,035	-0,107	0,300	0,183	0,414
**Clc1**	0,292	-0,044	0,018	-0,022	-0,148	-0,075	0,048	0,098	0,052	0,123	-0,118	0,080	0,301	-0,031	-0,534	-0,238	-0,009	0,184	0,182	0,017	0,022	0,217	0,329	-0,322	0,071	-0,249	-0,089
**Sur2a**	0,188	0,240	0,018	0,064	0,274	-0,093	0,180	-0,008	0,128	-0,306	0,267	-0,177	-0,168	-0,054	0,123	-0,305	0,083	0,068	0,218	-0,362	-0,046	-0,176	-0,052	-0,253	0,055	0,028	-0,376
**Sur2b**	0,236	-0,068	0,204	0,054	-0,320	0,261	-0,132	-0,015	-0,184	-0,341	0,163	-0,068	-0,002	0,144	0,223	-0,061	-0,378	0,214	-0,130	0,248	0,054	0,280	-0,165	-0,214	0,093	0,101	-0,109
**Ryr1**	0,223	0,238	0,000	0,309	-0,008	0,081	-0,088	0,035	-0,045	0,033	0,036	-0,112	-0,040	0,011	-0,277	0,008	0,140	-0,150	-0,504	0,342	0,090	-0,376	0,043	0,076	-0,082	-0,072	-0,324
**Nav1.4**	0,297	0,069	-0,075	0,195	-0,076	-0,085	0,011	-0,127	-0,057	-0,094	-0,025	0,163	0,074	0,364	0,060	-0,121	0,030	-0,073	0,055	-0,178	0,094	-0,091	-0,373	0,186	0,033	-0,558	0,308
**Serca2**	-0,192	0,301	-0,031	-0,160	0,039	-0,033	-0,083	0,376	-0,074	-0,107	-0,185	0,248	-0,176	0,146	-0,141	-0,457	-0,286	-0,128	0,083	0,065	0,207	0,046	-0,066	0,152	-0,317	0,136	-0,026
**Serca1**	0,301	0,055	-0,027	0,163	-0,040	0,168	0,120	-0,032	-0,083	-0,065	-0,032	-0,009	0,035	0,091	-0,191	0,217	-0,063	-0,359	-0,136	-0,478	-0,192	0,327	0,075	0,058	-0,326	0,297	0,035
**Irisin**	0,089	0,175	-0,474	-0,080	0,062	0,097	0,300	0,308	-0,120	0,014	0,070	-0,209	0,279	-0,024	0,042	0,009	-0,023	0,253	-0,226	-0,101	0,315	-0,020	-0,008	0,060	0,243	0,198	0,250
**Calcineurin**	0,105	0,351	-0,095	0,103	-0,066	-0,070	-0,054	-0,134	-0,230	-0,286	-0,053	-0,016	-0,017	0,105	0,095	-0,004	0,246	-0,089	0,346	0,296	-0,165	0,098	0,425	0,264	0,236	0,152	0,126
**Nmdar1**	0,109	-0,125	-0,226	-0,397	-0,141	-0,181	-0,458	-0,126	-0,287	0,026	-0,033	-0,343	-0,374	0,071	-0,169	-0,045	0,111	-0,062	-0,087	-0,221	0,138	0,013	0,004	-0,060	0,113	-0,012	-0,014
**Ampar2**	0,100	0,074	0,523	-0,186	-0,215	-0,060	-0,053	0,121	-0,175	0,003	-0,025	0,163	0,245	-0,173	0,265	-0,148	0,441	-0,061	-0,171	-0,208	0,245	-0,087	0,081	-0,028	-0,054	0,104	0,092
**Notch1**	0,184	-0,153	-0,101	-0,200	0,034	0,260	-0,183	0,113	-0,217	0,083	0,060	0,109	0,375	-0,095	-0,088	-0,037	-0,054	-0,260	0,330	0,047	-0,242	-0,335	-0,318	0,073	0,163	0,187	-0,171
**Taut**	-0,075	0,249	-0,191	0,158	-0,184	-0,321	-0,421	0,140	0,185	0,237	0,323	-0,191	0,347	-0,060	0,263	-0,023	-0,091	-0,105	0,031	-0,021	-0,093	0,186	0,002	-0,008	-0,192	-0,069	-0,076
**Ampk**	0,217	0,135	0,120	-0,037	0,315	0,262	-0,241	0,036	-0,015	0,157	-0,314	-0,058	0,016	-0,070	0,282	0,231	-0,321	0,064	0,110	-0,170	0,239	-0,032	0,307	0,161	0,043	-0,251	-0,187
**Murf1**	-0,007	-0,007	-0,052	0,169	0,261	0,261	-0,328	-0,093	-0,059	0,253	0,079	0,173	0,007	0,381	-0,006	-0,131	0,304	0,498	0,010	-0,068	-0,086	0,013	0,001	-0,055	-0,189	0,239	0,043
**Mhc2a**	-0,203	0,245	-0,087	-0,234	0,113	0,114	-0,124	-0,301	0,051	-0,286	0,212	0,195	0,229	0,129	-0,144	0,325	-0,025	-0,160	0,045	0,038	0,317	-0,126	0,075	-0,398	-0,139	-0,047	0,098
**Mhc2b**	0,256	-0,024	-0,230	0,072	0,154	-0,126	-0,031	-0,185	0,148	0,250	-0,043	0,470	-0,158	-0,098	0,214	-0,149	-0,009	-0,292	-0,154	0,112	0,179	0,211	-0,032	-0,220	0,315	0,208	-0,041
**Mhc2x**	-0,109	-0,119	0,215	0,218	0,371	-0,374	0,152	-0,049	-0,598	0,190	0,068	-0,159	0,138	0,174	-0,004	-0,008	-0,188	-0,129	0,040	0,079	0,101	0,018	0,014	-0,174	-0,013	0,048	0,014
**Mhc1**	-0,166	0,289	-0,115	-0,134	-0,185	0,102	0,183	0,284	-0,252	0,198	-0,158	0,142	-0,140	0,244	0,198	0,215	0,137	-0,043	-0,070	-0,042	-0,332	0,035	-0,034	-0,363	0,100	-0,237	-0,222
**Achr1**	-0,146	0,270	0,227	-0,269	0,320	-0,067	-0,082	-0,154	0,080	-0,084	-0,050	-0,029	0,238	0,058	-0,193	-0,054	0,013	0,045	-0,322	-0,038	-0,231	0,353	-0,227	0,230	0,341	-0,056	-0,136
**Ngf**	0,184	0,039	-0,103	-0,315	0,109	-0,018	0,099	-0,154	-0,249	0,044	0,361	0,239	-0,078	-0,271	0,086	-0,143	-0,206	0,148	-0,230	0,054	-0,344	-0,091	0,239	0,101	-0,257	-0,210	0,161
**Mstn**	0,241	0,021	0,105	-0,188	0,322	0,232	0,041	0,241	0,109	0,097	0,127	-0,305	-0,140	-0,044	0,032	0,003	0,279	-0,216	0,135	0,354	0,032	0,293	-0,160	-0,131	-0,193	-0,156	0,255
**eigenvalues**	**9,782**	**4,830**	**2,491**	**1,987**	**1,566**	**1,165**	**1,003**	**0,729**	**0,687**	**0,572**	**0,450**	**0,328**	**0,298**	**0,240**	**0,232**	**0,181**	**0,151**	**0,117**	**0,099**	**0,053**	**0,047**	**0,031**	**0,021**	**0,019**	**0,008**	**0,002**	**0,001**

Supplementary Table S2. Eigenvectors (Principal Components, one for gene and Eigenvalues (on last row). From the standardized gene expression dataset have been computed a total of 27 eigenpairs (eigenvector-eigenvalue) by eigendecomposition of the covariance matrix. Each eigenvector is a vector composed by 27 weights, one for target gene. More the weight is far from 0, more the gene is important in selected PC.

Linear Discriminant Coefficients	PC1	PC2	PC3
**MLC/SOD1^{G93A} vs strain-matched WT**	-0,486	0,341	-1,824
**MLC/SOD1^{G93A} vs SOD1^{G93A}**	0,979	-0,439	-0,230
**SOD1^{G93A} vs. strain-matched WT**	0,829	-0,586	-0,361

Supplementary Table S3. In the table are reported Linear Discriminant Coefficient (LDC) of the three LDA performed. LDCs represent the weights of each PC in order to separate along LD the two selected classes in examination.

A

MLC/SOD1^{G93A} vs. strain-matched WT	
**Gene Name**	**PCA-LDA loadings**
Nav1.4	0,016197
Ryr1	0,028059
Kir6.2	0,031744
Sur2a	0,041754
Notch1	0,043295
Serca1	0,078237
Murf1	0,095965
Ngf	0,112215
Pkc alpha	0,152873
Sur1	0,158498
Clc1	0,190573
Calcineurin	0,242497
Bk	0,244738
Achr1	0,250397
Serca2	0,252413
Ampk	0,278152
Mhc2b	0,286488
Mstn	0,301911
Nmdar1	0,317163
Mhc2a	0,340563
Mhc2x	0,379355
Mhc1	0,388337
Taut	0,47021
Sur2b	0,509636
Pkc theta	0,585247
Irisin	0,880613
Ampar2	0,978233

B

MLC/SOD1^{G93A} vs. SOD1^{G93A}	
**Gene Name**	**PCA-LDA loadings**
Murf1	0,008358
Calcineurin	0,029177
Ampar2	0,055067
Sur2a	0,074971
Mhc2x	0,103371
Ryr1	0,114192
Irisin	0,119189
Ampk	0,124894
Pkc theta	0,134571
Taut	0,138225
Kir6.2	0,15027
Ngf	0,187044
Pkc alpha	0,188678
Bk	0,196665
Mstn	0,20221
Nmdar1	0,213316
Sur2b	0,214038
Mhc1	0,263056
Notch1	0,27073
Sur1	0,275773
Serca1	0,27628
Nav1.4	0,277535
Mhc2a	0,286557
Clc1	0,300853
Serca2	0,312414
Achr1	0,313895
Mhc2b	0,314397

C

SOD1^{G93A} vs. strain-matched WT	
**Gene Name**	**PCA-LDA loadings**
Sur2a	0,009293
Murf1	0,017238
Ryr1	0,045818
Ampk	0,056811
Calcineurin	0,084018
Kir6.2	0,088671
Mhc2x	0,09772
Bk	0,135662
Taut	0,138503
Irisin	0,142213
Mstn	0,149257
Ampar2	0,149577
Sur2b	0,161877
Ngf	0,167174
Pkc alpha	0,217874
Serca1	0,226677
Nav1.4	0,232868
Pkc theta	0,240153
Nmdar1	0,245104
Clc1	0,261248
Mhc1	0,265516
Sur1	0,276927
Notch1	0,279006
Mhc2a	0,280721
Mhc2b	0,309599
Serca2	0,32376
Achr1	0,361438

Supplementary Table S4. Linear Discriminant gene loading weights of the three PCA-LDA performed. Absolute values have been reported and sorted in growing manner. Thus, on the bottom of each table are present the most discriminant genes. (A) PCA-LDA for the separation of MLC/SOD1^{G93A} vs. strain-matched WT; (B) MLC/SOD1^{G93A} vs. SOD1^{G93A}; (C) SOD1^{G93A} vs. strain-matched WT.

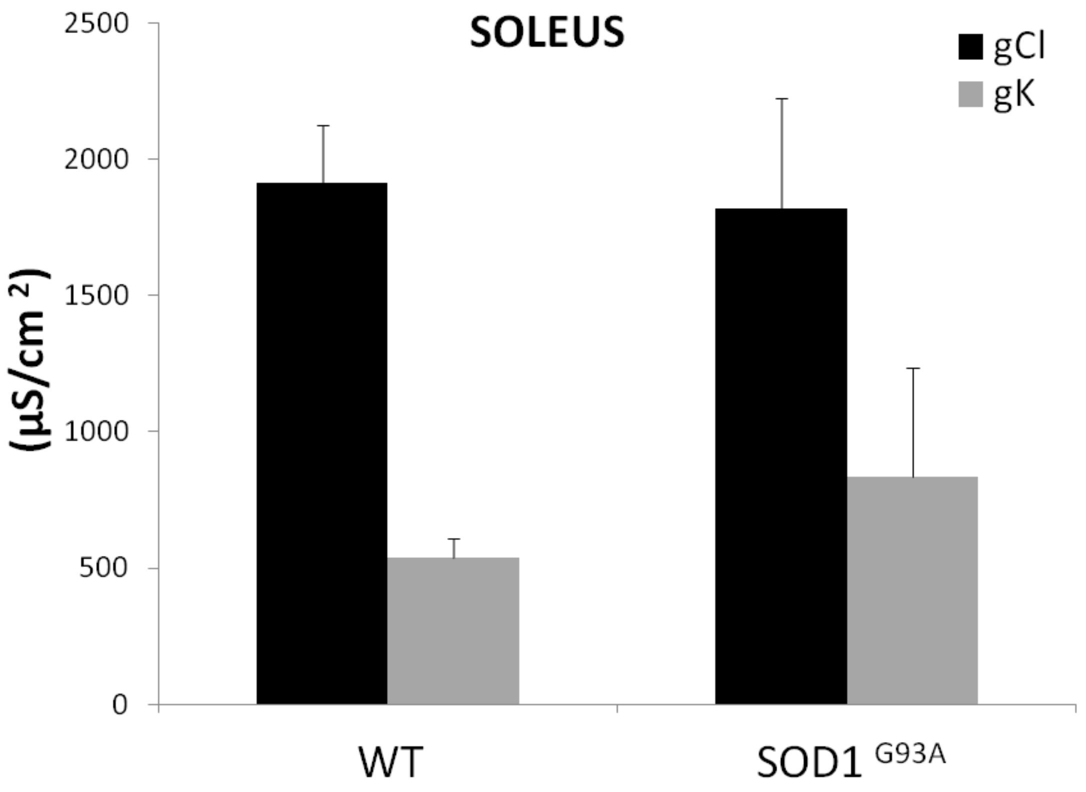

Supplementary Fig. S4. Resting chloride and potassium conductances in SOLEUS muscle of $SOD1^{G93A}$ transgenic mice. Resting Chloride (gCl) and Potassium (gK) conductances measurements have been done in slow-twitch SOLEUS muscles of $SOD1^{G93A}$ and WT at 130 days of age. As already demonstrated, the resting gCl in SOLEUS muscle of WT was significantly lower than in fast-twitch EDL muscles (Pierno et al., Brain, 125, 1510, 2002). In accord with the higher susceptibility of fast-fatigable motor-units, and lesser susceptibility of slow motor-units, no significant modifications have been detected in SOLEUS muscles of $SOD1^{G93A}$ mice. Values are expressed as mean ± S.E.M. from five animals for each experimental condition.

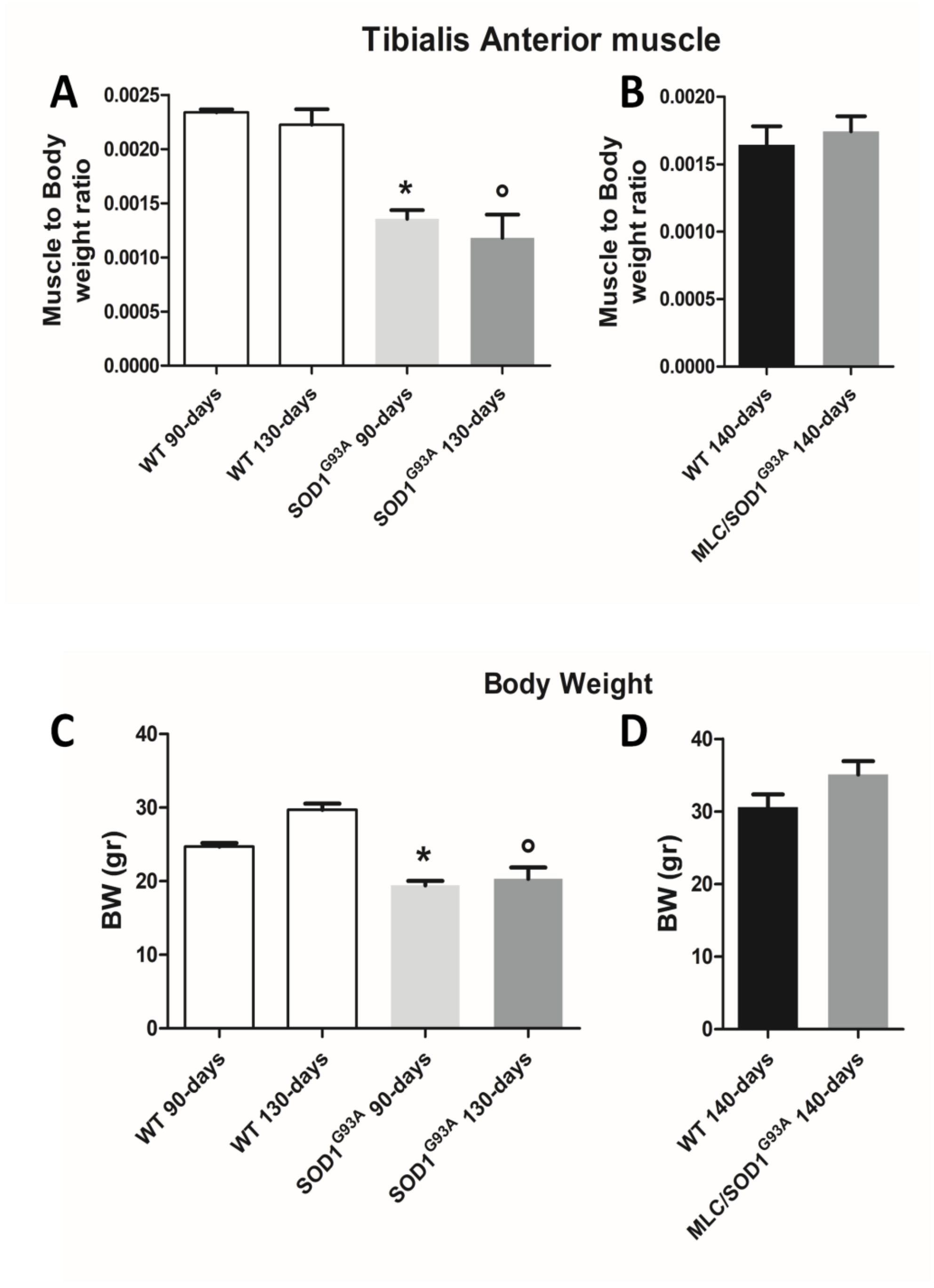

Supplementary Fig. S5. Skeletal muscle and body weight in $SOD1^{G93A}$ and in muscle specific $MLC/SOD1^{G93A}$ transgenic mice. Muscle to body weight ratio and body weight in $SOD1^{G93A}$ and $MLC/SOD1^{G93A}$ and age-matched wild-type (WT) mice. (A,B) Tibialis Anterior muscle weight was measured in each animal after dissection and normalized to body weight. Values are expressed as mean ± SEM from 5 animals for each experimental condition. (A) Statistical analysis was performed by two-ways ANOVA (Source of variation: interaction p=0.8284; GENOTYPE p<0.0001; AGE p=0.2995) followed by Bonferroni post-test. Significantly different (*) with respect to 90 days-old WT and (°) with respect to 130 days-old WT animals. (B) No significant differences were found between $MLC/SOD1^{G93A}$ and strain-matched WT mice (by Unpaired two-tailed Student-test). (C, D) Body weight was measured in 5 animals for each experimental condition. (C) Statistical analysis was performed by two-ways ANOVA (Source of variation: interaction p=0.0479; GENOTYPE p<0.0001; AGE p=0.0067) followed by Bonferroni post-test. Significantly different (*) with respect to 90 days-old WT and (°) with respect to 130 days-old WT animals. (D) No significant differences were found between $MLC/SOD1^{G93A}$ and strain-matched WT mice (by Unpaired two-tailed Student-test). The $SOD1^{G93A}$ genotype delays the body weight gain.

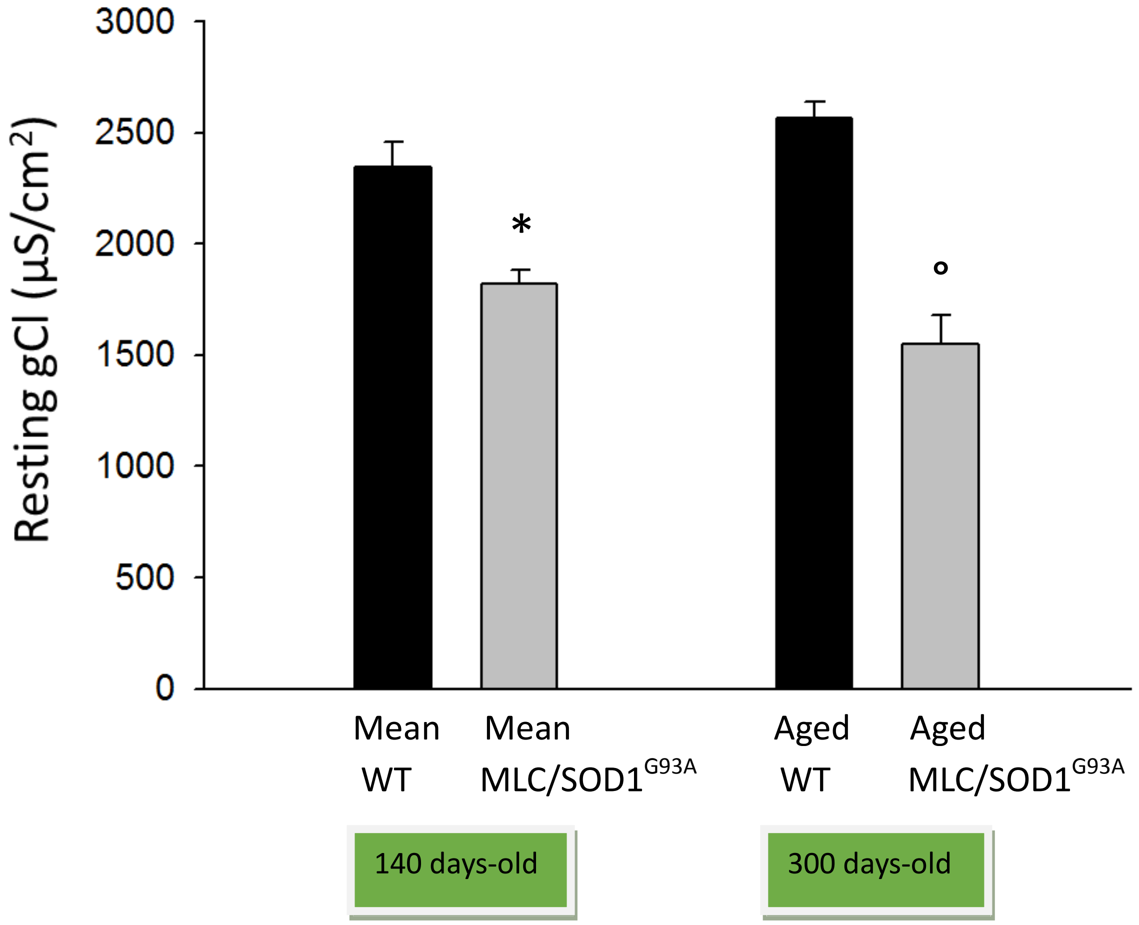

Supplementary Fig. S6. Resting chloride conductance in EDL muscle of 300-days-old MLC/SOD1^{G93A} mouse. Preliminary data showing the resting Chloride (gCl) conductance measured in EDL muscle of one 300-days-old MLC/SOD1^{G93A} mouse (Aged MLC/SOD1^{G93A}) compared to one age-matched control mouse (Aged WT). Further studies are in progress to evaluate aging process in MLC/SOD1^{G93A} animals. Values are expressed as mean ± S.E.M. from 5-7 fibers for each experimental condition. For comparison we reported here the mean values of gCl measured in 140 days-old animals, already shown in the manuscript. *Significantly different vs. mean WT (Student's t-test, P<0.001). °Significantly different vs. Aged WT (Student's t-test, P<0.001).

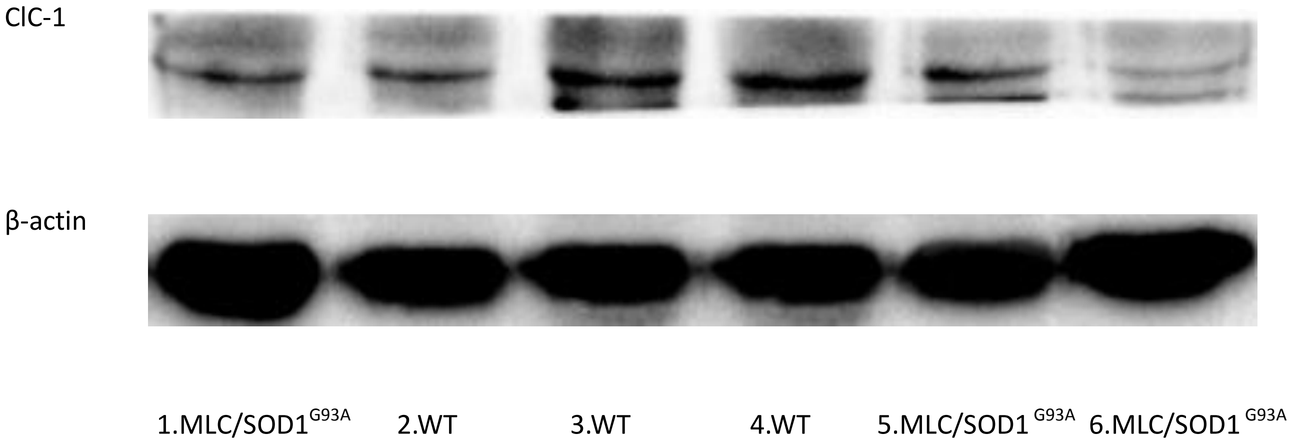

Supplementary Fig. S7. Full picture of the western blot, which is partially presented in Fig. 5 (4. and 5. are the cropped lines showed in the manuscript as example). ClC1 (75 - 150 kDa), β-actin (37 - 50 kDa).

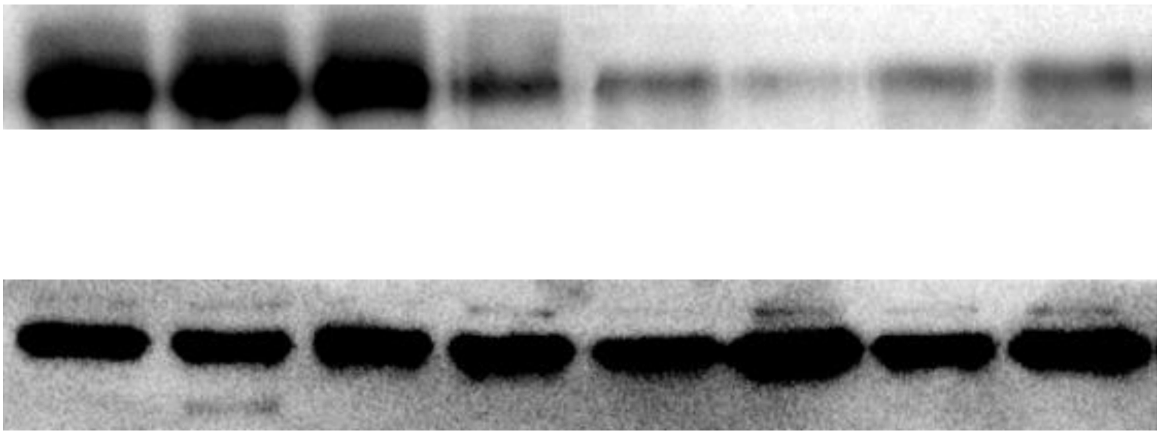

Supplementary Fig. S8. Full picture of the western blot, which is partially presented in Fig. 5 (3. and 4. are the cropped lines showed in the manuscript as example). ClC1 (75 - 150 kDa), β-actin (37 - 50 kDa).
